# Persistently increased CaMKIIδ autophosphorylation mediates pathologic SR Ca loss in a murine model of Doxorubicin-induced cardiomyopathy

**DOI:** 10.1016/j.jmccpl.2025.100828

**Published:** 2025-11-21

**Authors:** Anna-Lena Feder, Daniel Tarnowski, Anna-Maria Pfützenreuter, Maria Johanna Baier, Julian Mustroph, Maithily S. Nanadikar, Dörthe M. Katschinski, Lars Siegfried Maier, Can Martin Sag

**Affiliations:** a Department of Internal Medicine II/Cardiology, University Medical Center Regensburg, Germany; b Department of Pharmacology, Faculty of Medicine, University of Regensburg, Germany; c Institute of Cardiovascular Physiology, University Medical Center Göttingen, Georg August University, Göttingen, Germany

**Keywords:** Anthracycline, Cardiomyopathy, Heart failure, CaMKIIδ, ROS, SR Ca leak

## Abstract

**Background:**

Doxorubicin (DOX)-induced cardiomyopathy (DICM) manifests as left ventricular (LV) systolic dysfunction. DOX triggers oxidative stress and CaMKIIδ activity in cardiac myocytes. CaMKIIδ activation leads to impaired intracellular Ca handling and contractile dysfunction because of pathologic Ca loss from the sarcoplasmic reticulum (SR). While CaMKIIδ is canonically activated by autophosphorylation, it can also be activated via oxidation.

**Objectives:**

We aimed to investigate the *predominant* mode of CaMKIIδ activation in DICM.

**Methods:**

We utilized two transgenic mouse models, one lacking CaMKIIδ (CaMKIIδ^−/−^) and a “redox-dead” CaMKIIδ^Val281/282^ model. Acute changes in intracellular Ca handling and CaMKIIδ activation status were examined following 15 min of DOX exposure. Long-term effects were studied in CaMKIIδ^−/−^ mice (vs. CaMKIIδ^+/+^ wildtype littermates) and redox-dead CaMKIIδ^Val281/282^ mice (vs. CaMKIIδ^Met281/282^ wildtype littermates) that underwent DOX treatment in-vivo. Cardiac function (via echocardiography), intracellular Ca handling, and CaMKIIδ-related signaling were assessed 12 weeks post-treatment.

**Results:**

DOX *acutely* increased CaMKIIδ activity by autophosphorylation *and* oxidation in both WT lines, while autophosphorylated CaMKIIδ was still detected in CaMKIIδ^Val281/282^ mice, which resulted in comparably increased SR Ca leakage mediated by CaMKII-dependent RyR2-hyperphosphorylation at pS2814 in all aforementioned groups. In contrast, pharmacological and genetic inhibition of CaMKIIδ (i.e. in CaMKIIδ^−/−^) prevented DOX-induced CaMKIIδ-hyperactivation, RyR2-hyperphosphorylation and SR Ca loss. Similarly, only CaMKIIδ^−/−^ mice were protected from long-term DOX-induced LV dysfunction in-vivo. Redox-dead CaMKIIδ^Val281/282^ mice exhibited similar LV dysfunction as WT littermates, with persistent CaMKIIδ autophosphorylation, subsequent RyR2 hyperphosphorylation, and increased CaMKIIδ-dependent SR Ca leakage.

**Conclusions:**

Persistently increased CaMKIIδ autophosphorylation, but not oxidation, mediates pathologic SR Ca loss in Doxorubicin-induced cardiomyopathy.

## Graphical abstract

**Figure:**
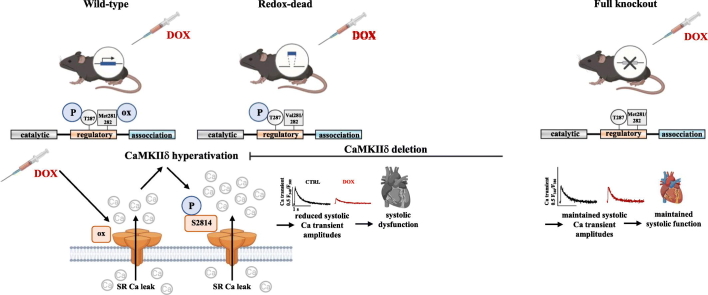

## 1 Introduction

Anthracyclines represent a well-established first-line therapy for the treatment of prevalent malignant diseases such as breast cancer [9]. Doxorubicin (DOX), a widely used anthracycline, is a well-known cause of dose-dependent left ventricular (LV) dysfunction with variable onset [19]. Cardinale et al. reported an overall incidence of 9 % for cardiotoxicity, defined as decrease in LV ejection fraction (EF) > 10 % that occurred in 98 % of cases within the first year after DOX treatment [5]. Despite the clinical need to develop targeted therapeutic strategies for DOX-induced cardiotoxicity (DICM) that preserve its oncological efficacy, the underlying pathomechanisms of DICM remain elusive. Among various hypotheses, DOX-induced reactive oxygen species (ROS) generation has been proposed as a key factor in DCIM development, linked to topoisomerase IIb inhibition, induction of apoptosis and Ca mishandling [4,6,17,21].

Impaired intracellular Ca handling has emerged as key pathological feature of DICM [18,25,28]. We have previously found that *acute* DOX exposure leads to severe Ca mishandling, characterized by reduced systolic Ca transient amplitudes due to CaMKII-dependent diastolic Ca loss from the sarcoplasmic reticulum (SR) [25], potentially contributing to contractile dysfunction observed clinically. In line with this, Llach et al. reported RyR2 hyperphosphorylation at the CaMKII specific site pS2814 leading to diastolic SR Ca loss in cardiac myocytes from DOX-treated mice, which was associated with long-term DOX-induced systolic dysfunction [18]. However, Llach et al. did not investigate the distinct mode of CaMKII activation, and whether CaMKII inhibition could protect from DOX-induced SR Ca loss and DOX-related LV dysfunction in-vivo.

It is well known that CaMKIIδ can be activated via phospho- or redox-dependent mechanisms [10]. Canonically, CaMKIIδ is activated by Ca/CaM-induced autophosphorylation at threonine-287, resulting in sustained CaMKIIδ activity. Alternatively, CaMKIIδ activity can be sustained by ROS-promoted oxidation of two redox-sensitive methionine residues within the autoregulatory domain of CaMKIIδ (referred to as Met281/282), which is associated with constitutive and autonomous CaMKIIδ activation [10]. However, it has not been investigated whether phospho- or redox-activated CaMKIIδ is predominantly required for the progression of DICM, and in turn, which strategy of CaMKII inhibition would be the most promising in treating DICM.

Here, we utilized two transgenic mouse models, one globally lacking CaMKIIδ (CaMKIIδ^−/−^) and a second mouse model, in which redox-dependent CaMKIIδ activation is inhibited by knock-in replacement of two oxidative-sensitive methionine residues in the regulatory domain with valine (CaMKIIδ^Val281/282^), to comprehensively investigate the predominant mode of CaMKIIδ activation and CaMKIIδ-related signaling during the progression of DICM.

## 2 Methods

### 2.1 Transgenic mouse models

All experiments were conducted in accordance with the guide for the care and use of laboratory animals and approved by the Institutional Animal Care and Use Committee and the District Government of Lower Franconia, Bavaria, Germany (AZ 55.2 2-532-2-1212-10). We used two transgenic mouse models to distinguish phospho- from redox-dependent CaMKIIδ activation in the course of DICM [2,10,18]. In a first model, CaMKIIδ was genetically eliminated (CaMKIIδ^−/−^) using a Cre/LoxP system [2]. CaMKIIδ^+/+^ littermates served as specific wildtype (WT) controls. In a second model, a “redox-dead” CaMKIIδ mouse model was used in which CaMKIIδ was made resistant to redox-dependent activation by knock-in replacement of two oxidative-sensitive methionine residues in the regulatory domain with valine (CaMKIIδ^Val281/282^) [10]. Here, redox-responsive WT littermates (CaMKIIδ^Met281/282^) served as specific controls. A third transgenic mouse model, in which CaMKII-dependent phosphorylation of RyR2 at serine-2814 is prevented by knock-in replacement of serine-2814 to alanine (S2814A) was used to verify the relevance of CaMKIIδ-dependent RyR2 phosphorylation [7].

### 2.2 Models of DICM

To investigate the *acute* effects of DOX, ventricular cardiomyocytes were isolated from 11 to 13 week old male mice and used for immediate in-vitro analysis as described below**.** DOX was used at 10 μmol/L, reflecting clinically relevant plasma concentrations [3].

The *long-term* effects of DOX were investigated in a widely used model of DICM, in which 11–13 week old male mice received three intraperitoneal (i.p.) injections of DOX (4 mg/kg) over the course of 14 days (on day 1, 7 and 14) or an equivalent volume of vehicle control (0.9 % NaCl) up to a total dose of 12 mg/kg [30]. Investigations were done after 12 ± 1 weeks.

### 2.3 Chemicals and pharmaceuticals

Doxorubicin was purchased from Sigma-Aldrich (44,583, St. Louis, MO, USA). If not indicated otherwise, Tyrode's solution was used as experimental solution containing (in mmol/L): NaCl 140, KCl 4, MgCl_2_ 5, HEPES 5, glucose 10, CaCl_2_ 1, pH 7.4.

In some experiments we used the scavenger Mito-TEMPO (25 nmol/L) (Sigma-Aldrich, St. Louis, MO, USA) to reduce mitochondrial superoxide formation. Stabilization of RyR closure and therefore pharmacological inhibition of SR Ca loss was ensured by incubating cardiomyocytes with the RyR inhibitor Dantrolene (1 μmol/L) (Sigma-Aldrich, St. Louis, MO, USA). CaMKII was pharmacologically inhibited by incubating cardiomyocytes with the CaMKII inhibitor AIP (1 μmol/L) (Sigma-Aldrich, St. Louis, MO, USA).

### 2.4 Echocardiography

Echocardiography was performed on anesthetized mice (isoflurane 1.5 %, spontaneous respiration) at baseline and after 12 ± 1 weeks. As described previously, the anterior chest of mice was shaved and mice were placed supine on a warming plate while body temperature was continuously monitored by a rectal probe to ensure constant body temperature (37 ± 1 °C) [15]. Echocardiographic recordings were performed in the parasternal short axis using Vevo LAB 3.0 Software. Two-dimensional echocardiographic images were acquired at papillary muscle level at a frame rate of about 300 Hz. M-mode images were obtained at a sweep speed of 100 mm/s. Images were analyzed using Vevo LAB 3.0. For this, the ejection fraction (LVEF), stroke volume (SV), cardiac output (CO), systolic volume (LVESV), diastolic volume (LVEDV), heart rate (HR), and dimensions of the left ventricle were assessed. LV dimensions included thicknesses of the septum (IVS), the posterior myocardial wall (PWTh), the inner diastolic (LVEDD) and systolic diameter (LVESD) of the left ventricle.

### 2.5 Mouse cardiac myocyte isolation

Ventricular cardiomyocytes were isolated as previously described using a Langendorff apparatus [13]. Explanted hearts were retrogradely perfused with (in mmol/L) NaCl 113, KCl 4.7, KH_2_PO_4_ 0.6, Na_2_HPO_4_x2H_2_O 0.6, MgSO_4_x7H_2_O 1.2, NaHCO_3_ 12, KHCO_3_ 10, HEPES 10, Taurine 30, 2,3-butanedione 10, glucose 5.5 and phenol red 0.032 at 37 °C (pH 7.4) for approximately 4 min. Afterwards, trypsin 0.6 % (Thermo Fisher Scientific Inc. of Waltham, MA, USA), liberase 7.5 mg/mL (Roche diagnostics, Mannheim, Germany) and CaCl_2_ 0.125 mmol/L were added to the perfusion solution to allow enzyme digestion of the extracellular matrix. The ventricular myocardium was then collected in perfusion buffer supplemented with 5 % bovine calf serum (Sigma-Aldrich, St. Louis, MO, USA), cut into small pieces and further disintegrated until no solid tissue was visible. At last, the cell suspension was filtered using a nylon gaze, before the cell suspension underwent a stepwise calcium reintroduction (from 0.1 to 0.8 mmol/L). Afterwards, isolated cardiomyocytes were plated on laminin-coated glass coverslips and cardiomyocytes were allowed to settle for 15 min to ensure cell adhesion on glass coverslips for subsequent functional measurements.

### 2.6 Isolation of ventricular human cardiomyocytes

All experiments were approved by local committees (ethics vote 22-2802_11–101) and are in accordance with the Helsinki Declaration. Written consent by patients had been given prior to tissue donation. Biopsies of ventricular myocardium were stored in ice-cold Custodiol solution containing butanedione monoxime (BDM, 2 mmol/L). Cardiomyocyte isolation was performed from vibratome-cut myocardial slices as described previously [11]. Briefly, myocardium samples were embedded into low-melting-point agarose and cut in 300 μm thick slices and enzymatically digested with proteinase XXIV (Sigma Aldrich, St. Louis, MO, USA) and collagenase type I (EMD Millipore Corp., Burlington, USA; 2 mg/mL). The digested tissue slices were dissociated with forceps by carefully pulling the fibers apart. For further experimental procedure, the cell suspension received a stepwise calcium reintroduction (to 1.5 mmol/L).

### 2.7 Epifluorescence microscopy

Intracellular Ca handling was assessed in isolated ventricular myocytes loaded with Fura2-AM (10 μmol/L, Thermo Fisher Scientific Inc. of Waltham, MA, USA) in the presence of 0.02 % (*w*/*v*) pluronic acid (Molecular Probes, Eugene, OR, USA) for 15 min at room temperature in darkness [13]. Afterwards, Fura2-AM loaded cardiomyocytes were deesterificated in Tyrode's solution for 15 min at room temperature in darkness. Upon deesterification, cells were plated on experimental chambers precoated with laminin and mounted on the stage of a Nikon Eclipse TE200-U inverted epifluorescence microscope (Ion Optix). Experiments were conducted during field-stimulation at 0.5 Hz and at 37 °C. Excitation of Fura2-AM at alternating wavelengths (F_340_ and F_380_, Ion Optix Hyperswitch System, IonOptix Corp., Westwood, MA, USA) resulted in emission that was collected at 510 nm for each wavelength. Ca transient amplitudes were calculated by the fluorescence ratio of F_340_/F_380_ after subtracting the background fluorescence at each excitation wavelength. SR Ca content was estimated by rapid application of caffeine (10 mmol/L, Sigma-Aldrich, St. Louis, MO, USA). Fluorescence emission was analyzed using Ion Wizard software (IonOptix Corporation, Boston, MA).

### 2.8 Confocal microscopy

Spontaneous diastolic SR Ca loss was assessed by measuring Ca spark frequency using a Zeiss LSM 700 confocal microscope microscope (Carl Zeiss AG, Oberkochen, Germany) [13]. Cardiomyocytes were loaded with Fluo4-AM (10 μmol/L, Thermo Fisher Scientific Inc. of Waltham, MA, USA) in presence of 0.02 % (*w*/*v*) pluronic acid (Molecular Probes, Eugene, OR, USA) for 15 min at room temperature in darkness. ROS formation was acutely assessed in cardiomyocytes loaded with CellROX Deep Red (5 μmol/L, Thermo Fisher Scientific Inc. of Waltham, MA, USA) for 30 min at 37 °C, which avoids the detection of DOX-induced autofluorescence typically present at about 590 nm. Chronic ROS formation was assessed in isolated cardiomyocytes loaded with CellROX Orange (5 μmol/L, Thermo Fisher Scientific Inc. of Waltham, MA, USA) for 30 min at 37 °C in darkness. Subsequently, dye-loaded cardiomyocytes were rinsed with Tyrode's solution and mounted on the laser scanning confocal microscope. Cardiomyocytes were stimulated at 0.5 Hz and superfused with Tyrodes's solution at 37 °C. Fluo4-loaded cardiomyocytes were excited via an argon laser (at 488 nm) and emitted fluorescence was collected after passing a 505 nm long-pass emission filter. Diastolic SR Ca release events (referred to as “Ca sparks”) were assessed using Zeiss Zen 3.1 software (10.000 lines per scan) upon termination of stimulation. Ca sparks were detected and quantified using Sparkmaster with manual detection of sparks. Ca spark frequency (CaSpF) was calculated and normalized to cell with and scanning time. Only cardiomyocytes that showed stimulated Ca transients were included in the evaluation to avoid misinterpretation as a consequence of an increased diastolic Ca leak in non-viable/dying cardiomyocytes. CellROX Deep Red-loaded cardiomyocytes were excited at 644 nm and fluorescence emission was collected at 665 nm. CellROX Orange-loaded cardiomyocytes were excited at 545 nm and fluorescence emission was collected at 565 nm. Fluorescence emission from both CellROX Deep Red and CellROX Orange-loaded cardiomyocytes was collected every minute over a time course of 15 min and was normalized to baseline fluorescence.

### 2.9 ELISA

#### 2.9.1 Detection of MDA and WST absorbance

Malondialdehyde (MDA) concentration and superoxide dismutase (SOD) activity were detected using the MDA assay kit (Signosis, Inc., USA), and the SOD Activity Assay Kit (Signosis, Inc., USA) according to manufactures instructions.

##### 2.9.1.1 MDA assay

Lipid peroxidation was detected by measuring MDA formation using thiobarbituric acid (TBA). In brief, tissue lysates were incubated with TBA for 1 h at 95 °C and then cooled at 4 °C for 10 min. Absorbance was measured at 532 nm using a plate reader. The distinct MDA concentration of a sample was calculated in relation to a standard curve that was generated using the MDA standards of the kit.

##### 2.9.1.2 SOD activity assay

SOD Activity was measured using a WST-based colorimetric assay. Briefly, the SOD Activity Assay utilizes WST to assess SOD activity. SOD can catalyze the reaction of superoxide anion radicals to form oxygen and hydrogen peroxide. In the SOD Activity Assay as used in our study, SOD activity is indirectly measured by using WST for the detection of superoxide levels. The interaction of WST with superoxide results in increased absorbance at 450 nm. Therefore, high levels of WST absorbance reflect suppressed SOD activity, whereas low levels of WST absorbance indicate increased SOD activity. To test this, lysates were incubated with WST and oxidase at 37 °C for 45 min. Afterwards, the absorbance was measured at 450 nm using a microplate reader.

### 2.10 Western blot

We used isolated cardiomyocytes acutely exposed to 10 μmol/L of DOX for 15 mins (vs. vehicle) and perfused cardiac tissue to assess the *acute* effects of doxorubicin. In addition, whole mouse heart tissue was taken from DOX treated mice and used to assess the *chronic* effects of doxorubicin. Samples were lysed in Tris buffer containing (in mmol/L): Tris-HCl 20, NaCl 200, NaF 20, Na_3_VO_4_ 1, Triton X 100 1 %, DTT 1, along with protease inhibitor cocktail and phosphatase inhibitor cocktail (both Roche Diagnostics, Mannheim, Germany). Samples were denatured for 5 min at 95 °C for CaMKIIδ and pT287/CaMKII, for 5 min at 95 °C under non-reducing conditions for oxCaMKII (i.e. in the absence of β-mercaptoethanol), and for 30 min at 37 °C for RyR2, pS2814/RyR2, SERCA2a, PLB, pT17/PLB. After denaturation, proteins were separated on a SDS-polyacrylamide gel (5 % SDS-polyacrylamide gel for detection of RyR2, pS2814/RyR2, pS2809/RyR2; 8 % SDS-polyacrylamide gel for detection of pT287/CaMKII, CaMKIIδ, oxCaMKII, SERCA2a and Catalase; 12.5 % SDS-polyacrylamide gel for detection of pT17/PLB, PLB), transferred to a nitrocellulose membrane and blocked with 5 % milk for one hour at room temperature. Afterwards, membranes were incubated with primary antibodies (diluted in 5 % milk): rabbit polyclonal anti-RyR2 (1:10000, Sigma Aldrich, St. Louis, MO, USA), rabbit polyclonal anti-pS2814/RyR2 (1:2000, Badrilla, Leeds, United Kingdom), rabbit polyclonal anti-pT17/PLB (1:10000, Badrilla, Leeds, United Kingdom), mouse monoclonal anti-PLB (1:20000, Millipore, Burlington, MA, USA), rabbit polyclonal anti-pT287/CaMKII (1:1000, PhosphoSolutions, Davis, CA, USA), rabbit polyclonal anti-CaMKIIδ (1:10000, Thermo Fisher Scientific Inc. of Waltham, MA, USA), rabbit polyclonal anti-oxCaMKII (1:1000, GeneTex, Irvine, CA, USA), rabbit polyclonal anti-Catalase (1:200, abcam, Cambridge, Great Britain), mouse monoclonal anti-SERCA2a (1:20000, Thermo Fisher Scientific Inc. of Waltham, MA, USA), and mouse monoclonal anti-GAPDH (1:10000, Sigma Aldrich, St. Louis, MO, USA) at 4 °C overnight. Secondary antibodies were HRP-conjugated sheep anti-mouse IgG (1:10000, GE Healthcare, Chicago, Illinois, USA) and anti-rabbit lgG (GE Healthcare, Chicago, Illinois, USA) and incubated for one hour at room temperature. For chemiluminescent detection, Immobilon™ Western Chemiluminescent HRP Substrate (Millipore, Burlington, MA, USA) was used. Values were normalized to GAPDH.

### 2.11 Statistical analysis

Experimental data are presented as mean ± SEM. The n number as shown in each figure/table depicts n = cells/mice or n = cells/human samples to show the number of technical replicates (number of cells) and biological replicates (number of mice or human samples). For statistical analysis Graph Pad Prism 9 as well as SigmaPlot 12.5 was used. Normality was tested using D'Agostino-Pearson test or Shapiro-Wilk test. Parametric (unpaired student's *t*-test as well as one-way (OW)/two-way (TW) analysis of variance (ANOVA) combined with Holm Sidak or Fisher LSD post-hoc test or non-parametric (Kruskal-Wallis, Mann-Whitney-U) tests were performed as indicated in the Figure legends. Values were considered statistically significant for *p*-values <0.05.

## 3 Results

### 3.1 CaMKIIδ autophosphorylation mediates DOX-induced SR Ca loss

**Fig. 1:**
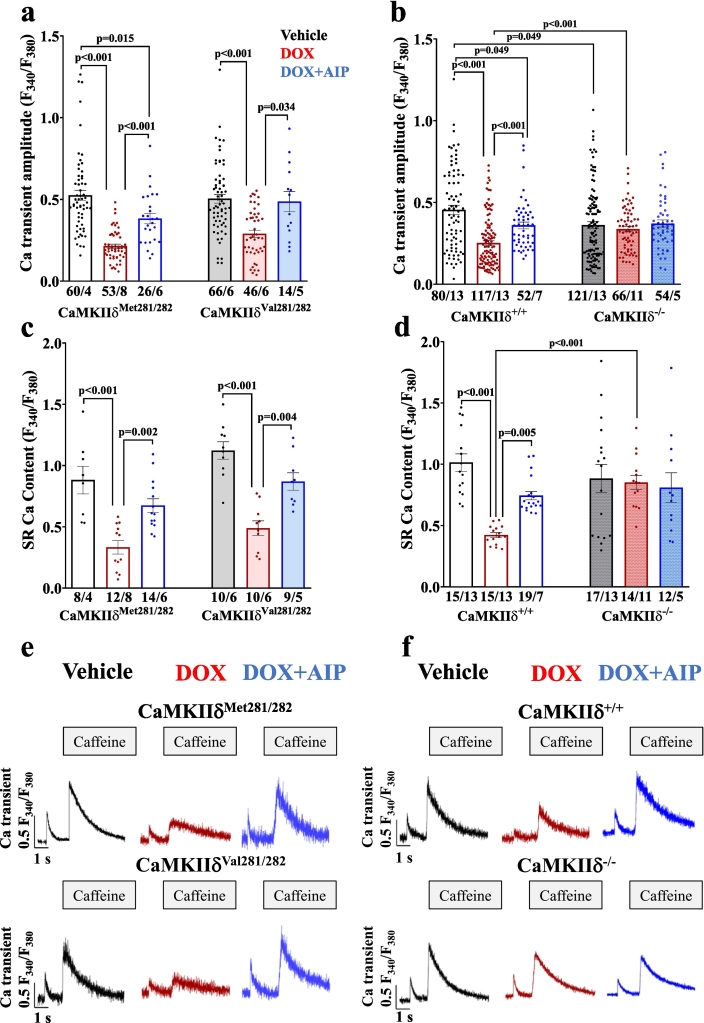
CaMKIIδ autophosphorylation reduces systolic Ca transients and SR Ca content upon acute DOX exposure. **a-**Reduced systolic Ca transient amplitudes upon acute DOX exposure in redox-sensitive CaMKIIδ^Met281/282^ (left) and redox-dead CaMKIIδ^Val281/282^ cardiomyocytes (right) that can be restored by pharmacological CaMKII inhibition using AIP (mean data). **b**-DOX-dependent impairment of systolic Ca transient amplitudes in CaMKIIδ^+/+^ cardiomyocytes (left) is absent upon pharmacological and genetic CaMKIIδ inhibition (right. Mean data). **c&d**-Mean data illustrate DOX-show reduction of SR Ca content in CaMKIIδ^Met281/282^ and CaMKIIδ^Val281/282^ cardiomyocytes (**c**), but not upon pharmacological CaMKII inhibition using AIP or in CaMKIIδ^−/−^ cardiomyocytes (**d**). **e&f-**Original traces of Fura-2 AM loaded cardiomyocytes illustrate reduced SR Ca content in CaMKIIδ^Met281/282^, CaMKIIδ^Val281/282^ (**e**) and CaMKIIδ^+/+^ cardiomyocytes. Cardiomyocytes with genetic deletion of CaMKIIδ (CaMKIIδ^−/−^) are protected from DOX-related SR Ca depletion (**f**). *P*-values were calculated using One-Way-ANOVA combined with Holm-Sidak post-hoc test (c) or Kruskal-Wallis test (a,b.d). n = cells/mice. DOX: doxorubicin; SR: sarcoplasmic reticulum.

**Fig. 2:**
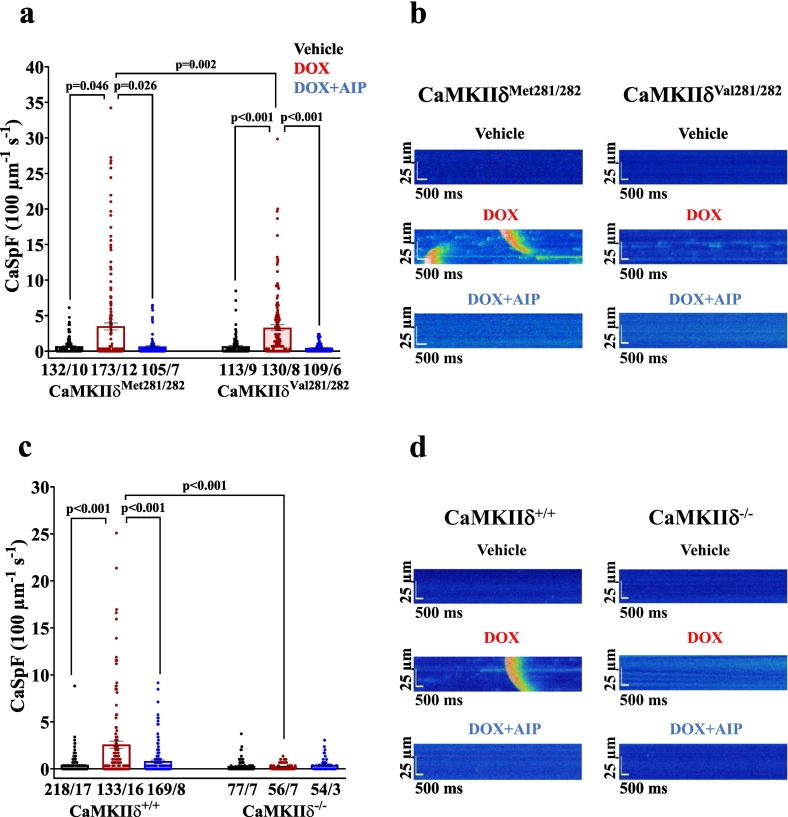
CaMKIIδ autophosphorylation mediates SR Ca loss upon acute DOX exposure. **a-**Mean data illustrate increased diastolic Ca spark frequency (CaSpF) in DOX-exposed CaMKIIδ^Met281/282^ and CaMKIIδ^Val281/282^ cardiomyocytes, which is prevented by pharmacological CaMKII inhibition (AIP). **b**-Original confocal line scan images of Fluo-4 AM loaded CaMKIIδ^Met281/282^ and CaMKIIδ^Val281/282^ cardiomyocytes. **c**-Diastolic CaSpF is absent upon genetic deletion of CaMKIIδ (CaMKIIδ^−/−^). **d**-Original confocal line scan images of Fluo-4 AM loaded CaMKIIδ^+/+^ and CaMKIIδ^−/−^ cardiomyocytes. *P*-values were calculated using Kruskal-Wallis test. n = cells/mice. CaSpF: calcium spark frequency; DOX: doxorubicin; SR: sarcoplasmic reticulum.

**Fig. 3:**
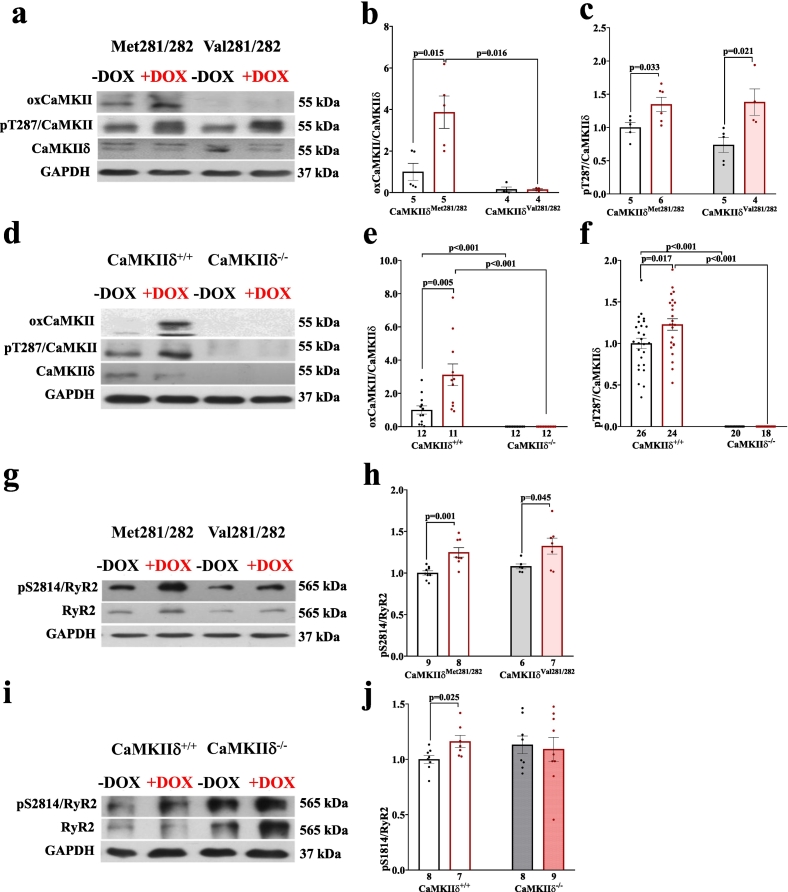
Increased CaMKIIδ autophosphorylation and CaMKIIδ-dependent RyR2-hyperphosphorylation at pS2814 upon acute DOX exposure. **a-c**- Original western blots (**a**) show increased CaMKII autophosphorylation at threonine-287 (pT287) (normalized to total CaMKIIδ expression) in DOX-exposed CaMKIIδ^Met281/282^ and CaMKIIδ^Val281/282^ cardiomyocytes (**c**), while CaMKII oxidation is prevented in CaMKIIδ^Val281/282^ (**b**). **d-f-** Original western blots (**d**) show increased CaMKII oxidation and autophosphorylation in CaMKIIδ^+/+^ while CaMKIIδ expression is deleted in CaMKIIδ^−/−^ cardiomyocytes (**e&f**). Original western blots (**g**) show CaMKII-specific hyperphosphorylation of pS2814 at the RyR2 in CaMKIIδ^Met281/282^ and CaMKIIδ^Val281/282^ cardiomyocytes (**h**), which is absent in CaMKIIδ^−/−^ cardiomyocytes (**i&j**). *P*-values were calculated using Mann-Whitney-*U* test (b) or unpaired Student's *t*-test (c,e,f,h,j). n = mice. DOX: doxorubicin; pS2814: CaMKII-specific RyR2 phosphorylation site at serine-2814; pT287: CaMKII autophosphorylation site at threonine-287; RyR2: ryanodine receptor.

Impaired intracellular Ca handling as acutely induced by DOX became evident by depressed systolic Ca transient amplitudes in DOX-treated WT cardiomyocytes (i.e. CaMKIIδ^Met281/282^ and CaMKIIδ^+/+^), as well as in redox-dead CaMKIIδ^Val281/282^ cells (Fig. 1**)**. This effect was associated with prolonged Ca transient decay (**Fig. S1 a&b**). Pharmacological inhibition of CaMKII (using the CaMKII-inhibitor AIP) in the aforementioned groups as well as genetic inhibition of CaMKIIδ (in CaMKIIδ^−/−^ cardiomyocytes) prevented this impairment in Ca handling upon acute DOX exposure. Similarly, SR Ca content was reduced in DOX-exposed CaMKIIδ^Met281/282^ and CaMKIIδ^+/+^, and in redox-dead CaMKIIδ^Val281/282^ cardiomyocytes, while pharmacological and genetic inhibition of CaMKIIδ maintained SR Ca content (Fig. 1). The reduction in SR Ca content in DOX-exposed CaMKIIδ^Met281/282^, CaMKIIδ^+/+^ and redox-dead CaMKIIδ^Val281/282^ cardiomyocytes was most likely due to increased pathologic diastolic Ca loss from the SR (Fig. 2). Both pharmacological and genetic inhibition of CaMKIIδ completely prevented DOX-dependent diastolic SR Ca loss, suggesting that CaMKIIδ autophosphorylation is crucial for mediating DOX-induced SR Ca loss. These functional observations were mirrored at the protein level, with DOX-dependently increased CaMKIIδ activity in CaMKIIδ^Met281/282^ and CaMKIIδ^+/+^ (by oxidation *and* autophosphorylation) as well as in redox-dead CaMKIIδ^Val281/282^ cardiomyocytes (by autophosphorylation *only*) (Fig. 3). Consequently, RyR2-hyperphosphorylation at the CaMKIIδ-specific site serine-2814 (pS2814) was present in all aforementioned groups (Fig. 3**,** Table 1 **a&b**). Vice versa, we did not observe RyR2-hyperphosphorylation at pS2814 in CaMKIIδ^−/−^ cardiomyocytes, which corroborates the crucial relevance of CaMKIIδ autophosphorylation in mediating DOX-related SR Ca loss in the acute setting.

**Table 1:** Protein expression and phosphorylation status of CaMKIIδ^Met281/282^, CaMKIIδ^Val281/282^ cardiomyocytes, CaMKIIδ^+/+^ and CaMKIIδ^−/−^ cardiomyocytes.

Table 1a								
Protein	CaMKIIδ^Met281/282^				CaMKIIδ^Val281/282^			
	Vehicle	n	DOX	n	Vehicle	n	DOX	n
CaMKIIδ	1.000 ± 0.084	5	1.265 ± 0.115	6	**0.717** **±** **0.078**^b^	5	**1.071** **±** **0.061**^a^	4
pT287/CaMKIIδ	1.000 ± 0.074	5	**1.346** **±** **0.108**^a^	6	0.737 ± 0.113	5	**1.380** **±** **0.199**^a^	4
oxCaMKII	1.000 ± 0.407	5	**3.871** **±** **0.781**^a^	5	0.154 ± 0.116	4	**0.143** **±** **0.039**^b^	4
PLB	1.000 ± 0.124	10	0.984 ± 0.104	10	0.934 ± 0.111	8	1.307 ± 0.495	8
pT17/PLB	1.000 ± 0.129	10	1.012 ± 0.136	10	1.057 ± 0.174	8	1.023 ± 0.144	8
RyR2	1.000 ± 0.062	9	0.964 ± 0.026	8	0.994 ± 0.090	6	0.798 ± 0.087	7
pS2814/RyR2	1.000 ± 0.025	9	**1.248** **±** **0.057**^a^	8	1.079 ± 0.030	6	**1.323** **±** **0.096**^a^	7
SERCA2a	1.000 ± 0.142	13	1.078 ± 0.092	14	1.054 ± 0.067	10	1.079 ± 0.060	10


Table 1b								
Protein	CaMKIIδ^+/+^				CaMKIIδ^−/−^			
	Vehicle	n	DOX	n	Vehicle	n	DOX	n
CaMKIIδ	1.000 ± 0.069	26	1.079 ± 0.089	24	n.d	20	n.d.	18
pT287/CaMKIIδ	1.000 ± 0.062	26	**1.228** **±** **0.069**^a^	24	n.d.	20	n.d.	18
oxCaMKII	1.000 ± 0.247	12	**3.117** **±** **0.655**^a^	11	n.d.	12	n.d.	12
PLB	1.000 ± 0.127	6	1.024 ± 0.129	6	1.136 ± 0.030	6	1.128 ± 0.093	6
pT17/PLB	1.000 ± 0.107	6	0.998 ± 0.108	6	**0.582** **±** **0.059**^b^	6	0.811 ± 0.140	6
RyR2	1.000 ± 0.119	8	1.165 ± 0.132	7	1.018 ± 0.181	8	1.123 ± 0.172	9
pS2814/RyR2	1.000 ± 0.036	8	**1.162** **±** **0.055**^a^	7	1.131 ± 0.080	8	1.092 ± 0.108	9
SERCA2a	1.000 ± 0.142	17	1.060 ± 0.138	17	1.191 ± 0.140	13	1.146 ± 0.129	15

CaMKIIδ^Met281/282^: wildtype; CaMKIIδ^Val281/282^: redox-dead; CaMKIIδ^+/+^: wildtype; CaMKIIδ^-/-^: genetic CaMKIIδ deletion; CaMKIIδ: Calcium/Calmodulin-dependent protein kinase II δ isoform; pT287/CaMKIIδ; CaMKII autophosphorylation site; oxCaMKII: oxidative-activated CaMKII; PLB: phospholamban; pT17/PLB: CaMKII-specific PLB phosphorylation site; RyR2: ryanodine receptor; pS2814/RyR2; CaMKII-specific RyR2 phosphorylation site; SERCA2a: SR Ca ATPase

a Indicates significance vs. vehicle.

b Indicates significance (P<0.05) between genotypes using Mann-Whitney-U-test or unpaired Student’s t-test. n=mice

In a next step, we aimed to investigate the mechanisms leading to CaMKIIδ autophosphorylation in the context of acute DOX treatment. Hyperactivation of CaMKIIδ by autophosphorylation requires increased levels of Ca-bound CaM, which may result from an initial pulse of Ca leaking out of the SR due to oxidized RyR2. DOX-related oxidation of the RyR2 may be a direct consequence of DOX oxidizing thiol-residues at the RyR [14,27] and/or an indirect effect of increased mitochondrial ROS-formation following DOX exposure [25]. Hence, we incubated DOX-treated WT cardiomyocytes with either Dantrolene (1 μmol/L) to stabilize the RyR2 despite DOX-related ROS-formation, or with Mito-TEMPO (25 nmol/L) to prevent DOX-related mitochondrial ROS-formation. DOX acutely increased cytoplasmic ROS formation in WT, CaMKIIδ^Val281/282^ and CaMKIIδ^−/−^ myocytes, which was attenuated by Mito-TEMPO in all genetic lines (**Fig. S2**). At the protein level, Mito-TEMPO and Dantrolene both prevented CaMKIIδ autophosphorylation in WT, which was in line with absent CaMKII-specific hyperphosphorylation of the RyR2 at pS2814 (**Fig. S3,** Table 2). In addition, DOX-related SR Ca loss was strongly inhibited in WT, CaMKIIδ^Val281/282^ and CaMKIIδ^−/−^ cardiomyocytes by both Mito-TEMPO and Dantrolene, which points to the fact that RyR2 *de*stabilization and mitochondrial ROS-formation are signaling events that that precede CaMKIIδ autophosphorylation upon acute DOX treatment. SERCA2a and PLB protein levels, as well as CaMKIIδ-dependent PLB phosphorylation (at pT17) were unchanged in CaMKIIδ^Met281/282^, CaMKIIδ^+/+^, CaMKIIδ^Val281/282^ and CaMKIIδ^−/−^ cardiomyocytes in the acute setting of DOX exposure (Table 1 **a&b**). Finally, we verified the relevance of CaMKII-dependent hyperphosphorylation of the RyR2 for the induction of DOX-dependent SR Ca loss in myocytes from a mouse model that is resistant to CaMKII-specific phosphorylation at serine-2814 by knock-in replacement of the CaMKII-specific phosphorylation site serine-2814 to alanine (S2814A model). As shown in **Fig. S4,** DOX-induced diastolic Ca loss from the SR was prevented in S2814A cardiomyocytes despite hyperactivation of CaMKIIδ (by both oxidation and autophosphorylation), which underscores the relevance of CaMKIIδ-mediated RyR2 hyperphosphorylation for the induction of DOX-dependent SR Ca loss (Table 3).

**Table 2:** Protein expression and phosphorylation status of WT cardiomyocytes upon Mito-TEMPO and Dantrolene.

Protein	WT							
	Vehicle	n	DOX	n	DOX+ MitoTEMPO	n	DOX+ Dantrolene	n
CaMKIIδ	1.000 ± 0.231	9	0.576 ± 0.096	13	0.967 ± 0.088	10	0.868 ± 0.079	10
pT287/CaMKIIδ	1.000 ± 0.132	9	**2.799** **±** **0.500**^a^	13	**1.219** **±** **0.168**^b^	10	**1.270** **±** **0.135**^b^	10
oxCaMKII	1.000 ± 0.248	7	**4.119** **±** **0.800**^a^	11	1.886 ± 0.655	7	2.146 ± 0.585	8
RyR2	1.000 ± 0.066	8	0.850 ± 0.090	7	0.945 ± 0.053	7	0.930 ± 0.068	7
pS2814/RyR2	1.000 ± 0.273	8	**3.268** **±** **0.655**^a^	7	**1.723** **±** **0.174**^b^	7	**1.093** **±** **0.183**^b^	7

CaMKIIδ: Calcium/Calmodulin-dependent protein kinase II d isoform; pT287/CaMKIIδ; CaMKII autophosphorylation site; oxCaMKII: oxidative-activated CaMKII; RyR2: ryanodine receptor; pS2814/RyR2; CaMKII-specific RyR2 phosphorylation site; WT: wildtype.

a Indicates significance vs. vehicle.

b Indicates significance vs. DOX using Kruskal-Wallis test or One-Way-ANOVA. n = mice.

**Table 3:** CaMKIIδ activation in WT and S2814A cardiomyocytes.

Protein	WT				S2814A			
	Vehicle	n	DOX	n	Vehicle	n	DOX	n
CaMKIIδ	1.000 ± 0.196	5	2.122 ± 0.459	6	0.976 ± 0.130	5	1.052 ± 0.427	4
pT287/CaMKIIδ	1.000 ± 0.227	6	**2.041** **±** **0.369**^a^	8	0.855 ± 0.213	5	**1.613** **±** **0.237**^a^	7
oxCaMKII	1.000 ± 0.054	8	**2.381** **±** **0.599**^a^	6	0.788 ± 0.085	7	**2.992** **±** **0.978**^a^	7

CaMKIIδ: Calcium/Calmodulin-dependent protein kinase II δ isoform; pT287/CaMKIIδ; CaMKII autophosphorylation site; oxCaMKII: oxidative-activated CaMKII.

a Indicates significance vs. Vehicle using unpaired Student's *t*-test. n = mice.

### 3.2 DOX-related LV-dysfunction is prevented in CaMKIIδ^-/-^ mice

**Fig. 4:**
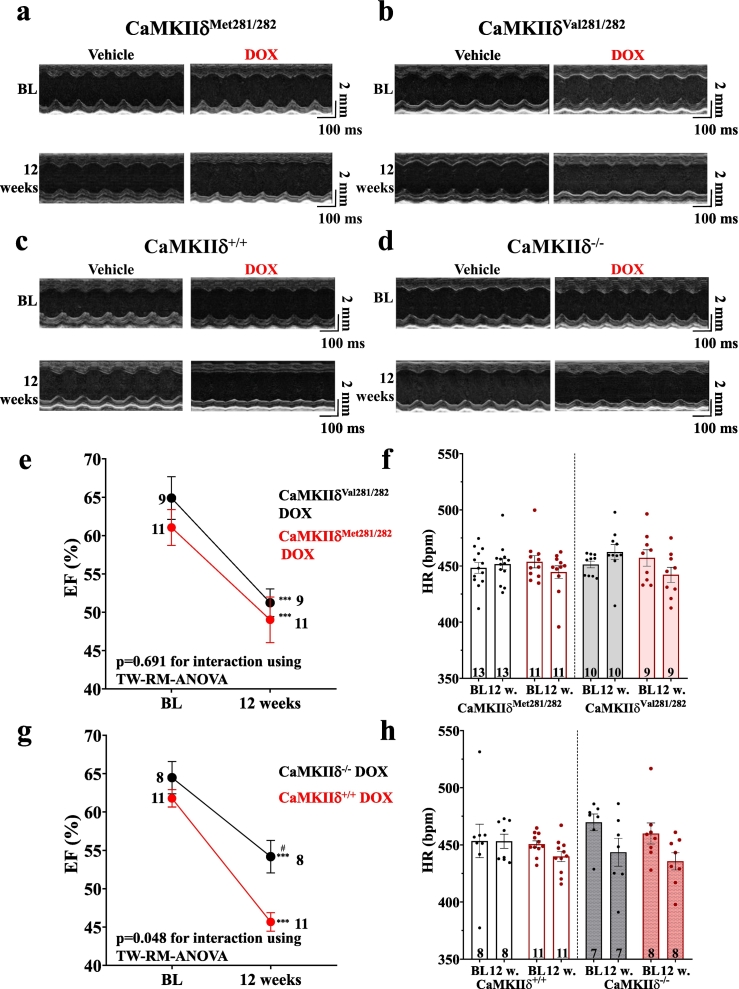
CaMKIIδ^−/−^ mice are protected from LV dysfunction in DICM. Original recordings of parasternal short axis at BL (upper panel) and after 12 weeks of DOX treatment (lower panel) in CaMKIIδ^Met281/282^ (**a**), CaMKIIδ^Val281/282^ (**b**), CaMKIIδ^+/+^ (**c**) and CaMKIIδ^−/−^ mice (**d**). **e**-Mean data illustrate that DOX-treated CaMKIIδ^Met281/282^ and CaMKIIδ^Val281/282^ mice develop comparably impaired left ventricular ejection fraction (EF).**f**-Mean data for heart rate (HR) in CaMKIIδ^Met281/282^ and CaMKIIδ^Val281/282^ mice. **g**-EF is reduced in DOX-treated CaMKIIδ^+/+^ and CaMKIIδ^−/−^ mice. However, CaMKIIδ^−/−^ mice are significantly protected upon DOX as compared to CaMKIIδ^+/+^ littermates. **h**-Mean data for HR during in CaMKIIδ^+/+^ and CaMKIIδ^−/−^ mice. *P*-values were calculated using mixed effect analysis combined with Fisher LSD post hoc test. n = mice. BL: baseline; DOX: doxorubicin; EF: left ventricular ejection fraction; HR: heart rate; 12w: 12 weeks. ^⁎^indicates significance vs. BL. ^#^indicates significance vs. genotype.

Next, we investigated the relevance of autophosphorylated vs. oxidized CaMKIIδ, and of CaMKIIδ-dependent SR Ca loss in the context of long-term DOX-induced cardiomyopathy in-vivo. As shown in **Fig. S5**, DOX-treated CaMKIIδ^Met281/282^, CaMKIIδ^+/+^, CaMKIIδ^Val281/282^ and CaMKIIδ^−/−^ mice all failed to gain body weight compared to vehicle-treated littermates, while heart weight remained unaffected. Furthermore, all lines exhibited impaired overall survival compared to vehicle-treated mice. This suggests a broadly comparable phenotype of DICM across genotypes, with no detectable survival advantage in any of the genetic strains (**Fig. S6**). Long-term DOX treatment led to a similar decrease of LVEF in CaMKIIδ^Met281/282^ (∼12 %) and redox-dead CaMKIIδ^Val281/282^ mice (∼14 %), with no significant difference between genotypes (Fig. 4**,** Table 4, Table 5, Table 6, Table 7). In contrast, DOX-treated CaMKIIδ^−/−^ mice showed the smallest reduction in LVEF (∼10 %), which was statistically significant compared to their specific CaMKIIδ^+/+^ littermates, where LVEF decreased by ∼16 % (*p* = 0.048 for interaction). Heart rates did not differ between groups.

**Table 4:** LV-function of CaMKIIδ^Met281/282^ mice upon long-term DOX treatment.

CaMKIIδ^Met281/282^					Interaction Vehicle vs. DOX
	Vehicle BL (n = 13)	Vehicle 12 W. (*n* = 13)	DOX BL (*n* = 11)	DOX 12 W. (*n* = 11)	
EF (%)	60.1 ± 1.2	58.3 ± 1.2	61.0 ± 2.3	**49.0** **±** **3.0**^a^^;^^b^	<0.001
FS (%)	31.6 ± 0.9	30.3 ± 0.8	32.5 ± 1.6	**24.6** **±** **1.9**^a^^;^^b^	<0.001
CO (mL/min)	17.2 ± 0.7	17.4 ± 1.1	19.0 ± 1.3	**13.8** **±** **0.8**^a^^;^^b^	=0.002
SV (μL)	38.4 ± 1.6	38.6 ± 2.4	41.8 ± 2.8	**31.0** **±** **1.7**^a^^;^^b^	=0.002
LVEDD (mm)	3.85 ± 0.07	3.89 ± 0.10	3.95 ± 0.10	3.85 ± 0.11	=0.345
LVEDV (μL)	64.3 ± 3.1	66.2 ± 4.2	68.7 ± 4.4	64.7 ± 4.2	=0.670
LVESD (mm)	2.64 ± 0.07	2.71 ± 0.0	2.67 ± 0.10	**2.91** **±** **0.13**^a^	=0.177
LVESV (μL)	25.8 ± 1.8	27.6 ± 2.1	26.9 ± 2.6	**33.7** **±** **3.6**^a^	=0.163
PWThd (mm)	0.84 ± 0.05	0.84 ± 0.07	0.83 ± 0.03	0.73 ± 0.02	=0.224
PWThs (mm)	1.08 ± 0.06	1.04 ± 0.06	1.08 ± 0.03	**0.91** **±** **0.05**^a^	=0.140
IVSd (mm)	1.17 ± 0.16	1.05 ± 0.06	1.02 ± 0.04	0.99 ± 0.06	=0.559
IVSs (mm)	1.44 ± 0.23	1.23 ± 0.05	1.22 ± 0.06	1.11 ± 0.08	=0.715
HR (bpm)	448 ± 5	452 ± 5	454 ± 5	445 ± 6	=0.229

BL: baseline; CaMKIIδ^Met281/282^: wildtype; CO: cardiac output; EF: ejection fraction; FS: fractional shortening; HR: heart rate; IVSd: end-diastolic interventricular septum; IVSs: end-systolic interventricular septum; LV: left ventricle; LVEDD: left ventricular end diastolic diameter; LVEDV: left ventricular end diastolic volume; LVESD: left ventricular end systolic diameter; LVESV: left ventricular end systolic volume; PWThd: posterior wall thickness, diastolic; PWThs: posterior wall thickness, systolic; SV: stroke volume; 12 W.: 12 weeks.

a Indicates significance vs. BL.

b Indicates significance vs. Vehicle using TW-RM-ANOVA. n = mice.

**Table 5:** LV-function of CaMKIIδ^Val281/282^ mice.

CaMKIIδ^Val281/282^					Interaction vehicle vs. DOX	Interaction CaMKIIδ^Val281/282^ DOX vs. CaMKIIδ^Met281/282^ DOX
	Vehicle BL (n = 10)	Vehicle 12 W. (*n* = 10)	DOX BL (n = 9)	DOX 12 W. (*n* = 9)		
EF (%)	62.4 ± 2.4	59.7 ± 2.4	64.9 ± 2.8	**51.2** **±** **1.8**^a^^;^^b^	=0.007	=0.691
FS (%)	33.2 ± 1.7	31.4 ± 1.7	35.2 ± 2.1	**25.7** **±** **1.1**^a^^;^^b^	=0.007	=0.550
CO (mL/min)	15.0 ± 0.7	17.5 ± 0.852	17.4 ± 0.7	**13.1** **±** **1.5**^a^^;^^b^	=0.003	=0.642
SV (μL)	32.3 ± 1.4	37.7 ± 1.7	38.1 ± 1.5	**30.1** **±** **3.0**^a^^;^^b^	=0.159	=0.490
LVEDD (mm)	3.58 ± 0.09^c^	3.84 ± 0.09	3.73 ± 0.10	3.68 ± 0.17	=0.132	=0.814
LVEDV (μL)	54.0 ± 3.1	63.9 ± 3.4	59.7 ± 3.7	58.8 ± 5.6	=0.145	=0.940
LVESD (mm)	2.40 ± 0.11	2.64 ± 0.11	2.43 ± 0.13	**2.73** **±** **0.13**^a^	=0.732	=0.763
LVESV (μL)	20.8 ± 2.2	26.2 ± 2.5	21.6 ± 2.8	**28.6** **±** **3.0**^a^	=0.688	=0.957
PWThd (mm)	0.81 ± 0.04	0.84 ± 0.04	0.80 ± 0.04	0.81 ± 0.07	=0.762	=0.200
PWThs (mm)	1.07 ± 0.07	1.01 ± 0.06	1.10 ± 0.04	0.98 ± 0.06	=0.558	=0.565
IVSd (mm)	0.97 ± 0.04	1.01 ± 0.05	1.02 ± 0.04	1.02 ± 0.05	=0.684	=0.792
IVSs (mm)	1.23 ± 0.10	1.18 ± 0.06	1.27 ± 0.07	1.12 ± 0.05	=0.391	=0.740
HR (bpm)	451 ± 3	463 ± 7	457 ± 7	442 ± 7	=0.055	=0.626

BL: baseline; CaMKIIδ^Met281/282^: wildtype; CaMKIIδ^Val281/282^: redox-dead; CO: cardiac output; EF: ejection fraction; FS: fractional shortening; HR: heart rate; IVSd: end-diastolic interventricular septum; IVSs: end-systolic interventricular septum; LV: left ventricle; LVEDD: left ventricular end diastolic diameter; LVEDV: left ventricular end diastolic volume; LVESD: left ventricular end systolic diameter; LVESV: left ventricular end systolic volume; PWThd: posterior wall thickness, diastolic; PWThs: posterior wall thickness, systolic; SV: stroke volume; 12 W.: 12 weeks.

a Indicates significance vs. BL.

b Indicates significance vs. Vehicle.

c Indicates significance between genotypes using TW-RM-ANOVA. n = mice.

**Table 6:** LV-function of CaMKIIδ^+/+^ mice.

CaMKIIδ^+/+^					Interaction Vehicle vs. DOX
	Vehicle BL (*n* = 8)	Vehicle 12 W. (n = 8)	DOX BL (n = 11)	DOX 12 W. (n = 11)	
EF (%)	57.8 ± 1.5	**52.8** **±** **1.5**^a^	**61.7** **±** **1.3**^b^	**45.8** **±** **1.3**^a^^;^^b^	<0.001
FS (%)	28.3 ± 0.9	**26.9** **±** **1.0**^a^	**32.9** **±** **0.9**^b^	**22.0** **±** **0.9**^a^^;^^b^	<0.001
CO (mL/min)	15.7 ± 0.9	17.2 ± 0.9	18.2 ± 0.8	**13.7** **±** **0.8**^a^^;^^b^	<0.001
SV (μL)	34.7 ± 2.4	**38.1** **±** **2.1**^a^	**41.6** **±** **1.8**^b^	**32.5** **±** **1.4**^a^^;^^b^	<0.001
LVEDD (mm)	3.75 ± 0.11	**4.05** **±** **0.11**^a^	3.90 ± 0.09	3.99 ± 0.10	=0.084
LVEDV (μL)	60.6 ± 4.5	**72.6** **±** **4.5**^a^	66.4 ± 3.8	70.2 ± 3.8	=0.100
LVESD (mm)	2.64 ± 0.11	**2.96** **±** **0.11**^a^	2.64 ± 0.09	**3.09** **±** **0.09**^a^	=0.125
LVESV (μL)	25.9 ± 2.8	**34.5** **±** **2.8**^a^	25.4 ± 2.4	**38.5** **±** **2.4**^a^	=0.220
PWThd (mm)	0.85 ± 0.04	0.79 ± 0.04	0.77 ± 0.03	0.74 ± 0.03	=0.767
PWThs (mm)	1.08 ± 0.04	0.95 ± 0.04^a^	1.04 ± 0.03	**0.89** **±** **0.03**^a^	=0.786
IVSd (mm)	1.05 ± 0.04	0.97 ± 0.04	0.97 ± 0.04	0.92 ± 0.04	=0.736
IVSs (mm)	1.27 ± 0.05	**1.12** **±** **0.06**^a^	**1.10** **±** **0.05**^b^	1.08 ± 0.05	=0.138
HR (bpm)	453 ± 8	453 ± 8	449 ± 7	438 ± 7	=0.494

BL: baseline; CaMKIIδ^+/+^: wildtype; CO: cardiac output; EF: ejection fraction; FS: fractional shortening; HR: heart rate; IVSd: end-diastolic interventricular septum; IVSs: end-systolic interventricular septum; LV: left ventricle; LVEDD: left ventricular end diastolic diameter; LVEDV: left ventricular end diastolic volume; LVESD: left ventricular end systolic diameter; LVESV: left ventricular end systolic volume; PWThd: posterior wall thickness, diastolic; PWThs: posterior wall thickness, systolic; SV: stroke volume; 12 W.: 12 weeks.

a Indicates significance vs. BL.

b Indicates significance vs. vehicle using TW-RM-ANOVA. n = mice.

**Table 7:** LV-function of CaMKIIδ^−/−^ mice.

CaMKIIδ^−/−^					Interaction Vehicle vs. DOX	Interaction CaMKIIδ^−/−^ DOX vs. CaMKIIδ^+/+^ DOX
	Vehicle BL (n = 7)	Vehicle 12 W. (*n* = 7)	DOX BL (n = 9)	DOX 12 W. (n = 9)		
EF (%)	**68.6** **±** **1.0**^c^	**64.1** **±** **0.5**^a^^;^^c^	64.5 ± 2.1	**54.2** **±** **2.1**^a^^;^^b^^;^^c^	=0.035	=0.048
FS (%)	**37.4** **±** **0.7**^c^	**34.0** **±** **0.3**^a^^;^^c^	34.6 ± 1.5	**27.4** **±** **1.4**^a^^;^^b^^;^^c^	=0.045	=0.048
CO (mL/min)	14.9 ± 1.3	14.7 ± 1.4	**15.5 ± 0.6**^c^	11.7 ± 0.8	=0.088	=0.528
SV (μL)	31.7 ± 2.7	33.1 ± 2.7	33.8 ± 1.5	27.0 ± 1.7	=0.075	=0.327
LVEDD (mm)	**3.35** **±** **0.13**^c^	**3.51** **±** **0.14**^c^	**3.55** **±** **0.09**^c^	**3.47** **±** **0.11**^c^	=0.298	=0.249
LVEDV (μL)	**46.3** **±** **4.0**^c^	**51.9** **±** **4.5**^c^	**53.0** **±** **3.3**^c^	**50.2** **±** **3.5**^c^	=0.276	=0.247
LVESD (mm)	**2.10** **±** **0.09**^c^	**2.32** **±** **0.10**^c^	**2.33** **±** **0.11**^c^	**2.52** **±** **0.10**^c^	=0.883	=0.045
LVESV (μL)	**14.6** **±** **1.4**^c^	**18.8** **±** **1.8**^c^	19.1 ± 2.2	**23.3** **±** **2.2**^c^	=0.990	=0.043
PWThd (mm)	0.84 ± 0.04	0.85 ± 0.06	0.84 ± 0.05	0.76 ± 0.03	=0.297	=0.485
PWThs (mm)	1.03 ± 0.04	1.04 ± 0.06	1.11 ± 0.09	0.96 ± 0.06	=0.245	=0.869
IVSd (mm)	1.08 ± 0.08	1.02 ± 0.05	1.05 ± 0.06	**1.03** **±** **0.04**^c^	=0.727	=0.667
IVSs (mm)	1.37 ± 0.07	**1.11** **±** **0.05**^a^	**1.17** **±** **0.05**^b^	1.14 ± 0.05	=0.034	=0.922
HR (bpm)	470 ± 7	444 ± 12	460 ± 9	436 ± 8	=0.939	=0.755

BL: baseline; CaMKIIδ^+/+^: wildtype; CaMKIIδ^−/−^: genetic CaMKIIδ deletion; CO: cardiac output; EF: ejection fraction; FS: fractional shortening; HR: heart rate; IVSd: end-diastolic interventricular septum; IVSs: end-systolic interventricular septum; LV: left ventricle; LVEDD: left ventricular end diastolic diameter; LVEDV: left ventricular end diastolic volume; LVESD: left ventricular end systolic diameter; LVESV: left ventricular end systolic volume; PWThd: posterior wall thickness, diastolic; PWThs: posterior wall thickness, systolic; SV: stroke volume; 12 W.: 12 weeks.

a Indicates significance vs. BL.

b Indicates significance vs. vehicle.

c Indicates significance between genotypes using TW-RM-ANOVA. n = mice.

### 3.3 Persistently increased CaMKIIδ autophosphorylation leads to pathologic SR Ca loss in DICM

**Fig. 5:**
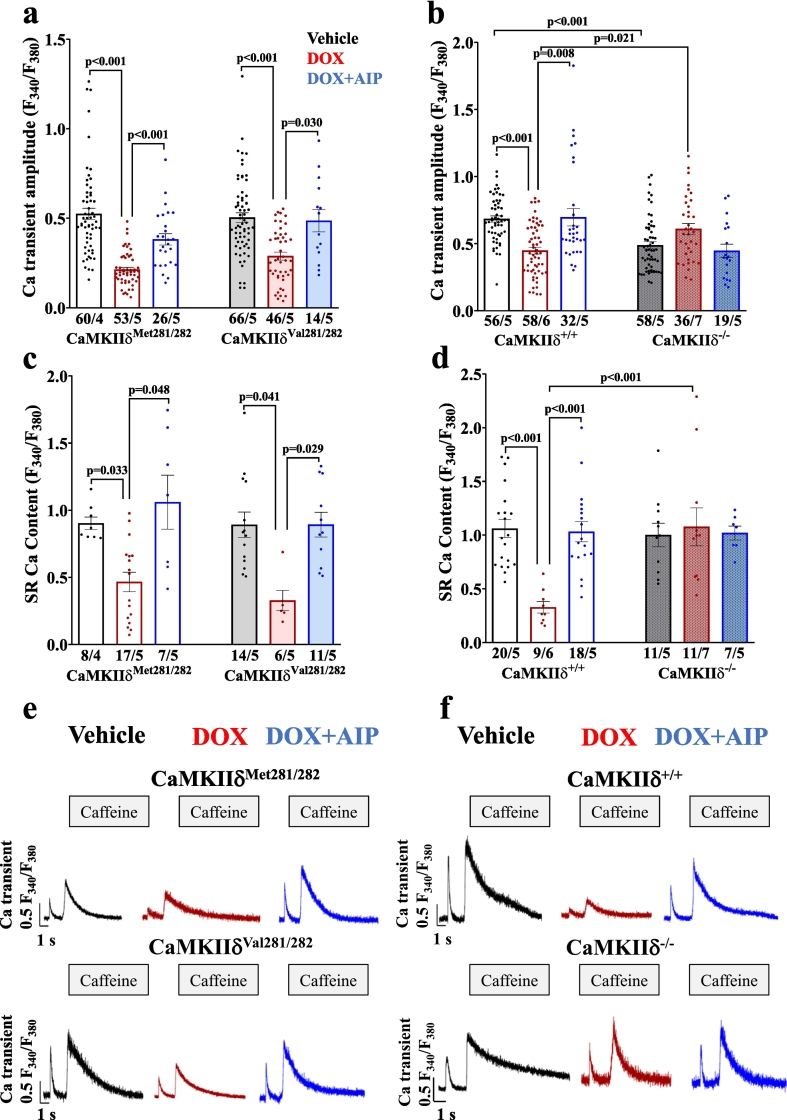
CaMKIIδ autophosphorylation cause reduced systolic Ca transients and SR Ca depletion in DICM. **a-**Reduced systolic Ca transient amplitudes in DICM as evident in CaMKIIδ^Met281/282^ and CaMKIIδ^Val281/282^ myocytes can be restored by pharmacological inhibition of CaMKII (AIP). **b**-Reduced Ca transient amplitudes in DICM as evident in CaMKIIδ^+/+^ myocytes are not present upon pharmacological CaMKII inhibition in CaMKIIδ^+/+^, and following genetic deletion of CaMKIIδ in CaMKIIδ^−/−^. **c-**Long-term DOX treatment results in SR Ca depletion in CaMKIIδ^Met281/282^ and CaMKIIδ^Val281/282^ myocytes that can be rescued by pharmacological inhibition of CaMKII. **d-**Genetic deletion of CaMKIIδ prevents DOX-induced SR Ca depletion. **e-**Original traces of caffeine-induced Ca transients demonstrate reduced SR Ca content in Fura-2 AM loaded CaMKIIδ^Met281/282^ and CaMKIIδ^Val281/282^ myocytes. **f-**Original traces of caffeine-induced Ca transients demonstrate maintained SR Ca content in DOX-treated CaMKIIδ^−/−^ myocytes. *P*-values were calculated using Kruskal-Wallis test (a,b,c) or One-Way-ANOVA combined with Holm-Sidak post-hoc test (d). n = cells/mice. DOX: doxorubicin; SR: sarcoplasmic reticulum.

**Fig. 6:**
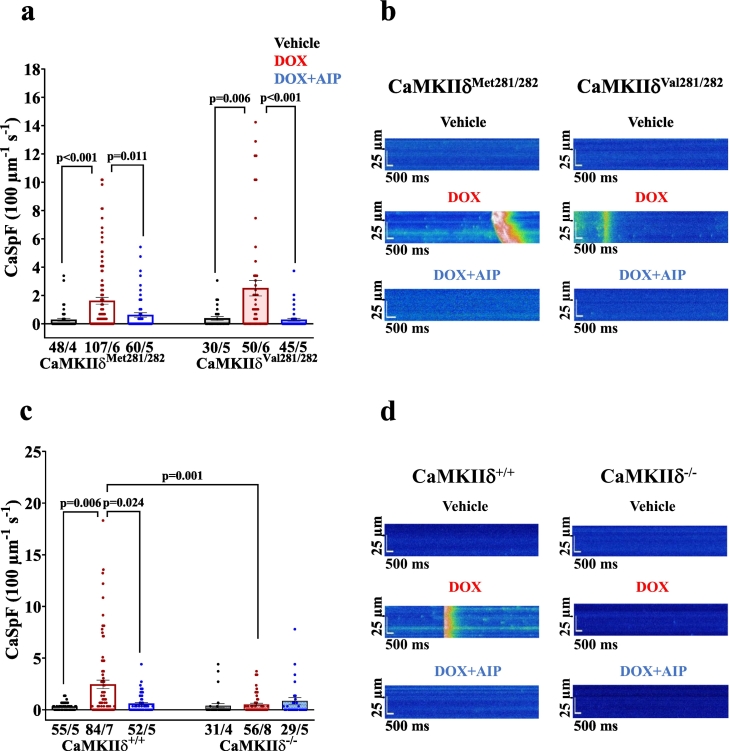
CaMKIIδ autophosphorylation mediates SR Ca loss in DICM. **a-**Increased diastolic Ca spark frequency (CaSpF) in DOX-treated CaMKIIδ^Met281/282^ and CaMKIIδ^Val281/282^ myocytes, which is abolished by pharmacological CaMKII inhibition (AIP). **b**-Original confocal line scan images of Fluo-4 AM loaded CaMKIIδ^Met281/282^ and CaMKIIδ^Val281/282^ cardiomyocytes. **c-**CaMKIIδ^+/+^ myocytes exhibit increased CaSpF upon DOX, which is not present in CaMKIIδ^−/−^ myocytes or upon pharmacological CaMKII inhibition (AIP). **d**-Original confocal line scan images of Fluo-4 AM loaded CaMKIIδ^+/+^ and CaMKIIδ^−/−^ cardiomyocytes. *P*-values were calculated using Kruskal-Wallis test. n = cells/mice. CaSpF: calcium spark frequency; DOX: doxorubicin;

**Fig. 7:**
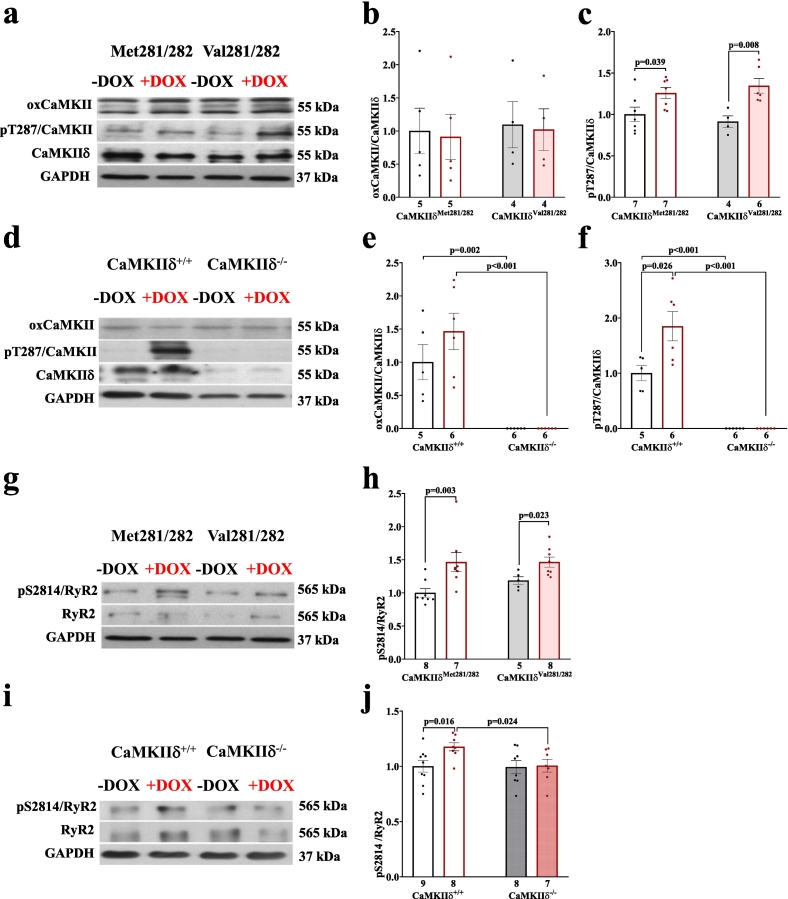
Persistently increased CaMKIIδ autophosphorylation and CaMKIIδ-dependent RyR2-hyperphosphorylation at pS2814 in DICM. **a-c**- Original western blots (**a**) show persistently increased CaMKIIδ autophosphorylation at threonine-287 (pT287) (normalized to total CaMKIIδ expression) in CaMKIIδ^Met281/282^ and CaMKIIδ^Val281/282^ cardiomyocytes in DICM (**c**), while CaMKIIδ oxidation is unaltered (**b**). Original western blots (**d**) show increased CaMKIIδ autophosphorylation in CaMKIIδ^+/+^ while CaMKIIδ expression is deleted in CaMKIIδ^−/−^ cardiomyocytes (**f**). Original western blots (**g**) show persistent CaMKII-specific hyperphosphorylation of pS2814 at the RyR2 in CaMKIIδ^Met281/282^ and CaMKIIδ^Val281/282^ cardiomyocytes in DICM (**h**), which is absent in CaMKIIδ^−/−^ cardiomyocytes (**i&j**). *P*-Values were calculated using unpaired Student's t-test (b,c,e,f,j) or Mann-Whitney-U test (h). n = mice. DOX: doxorubicin; pS2814: CaMKII-specific RyR2 phosphorylation site at serine-2814; pT287: CaMKII autophosphorylation site at threonine-287; RyR2: ryanodine receptor.

Consistent with impaired LVEF observed in-vivo, cardiomyocytes from long-term DOX-treated CaMKIIδ^Met281/282^, CaMKIIδ^+/+^, and redox-dead CaMKIIδ^Val281/282^ mice revealed reduced Ca transient amplitudes (Fig. 5), which were associated with prolonged decay kinetics (**Fig. S1c&d**). In contrast, Ca handling remained largely preserved in cardiomyocytes from long-term DOX-treated CaMKIIδ^−/−^ mice. Vice versa, pharmacological inhibition of CaMKII with AIP restored Ca transients in cardiomyocytes from CaMKIIδ^Met281/282^, CaMKIIδ^+/+^, and redox-dead CaMKIIδ^Val281/282^ mice following in-vivo DOX treatment. SR Ca content was found to be reduced following long-term DOX treatment in cardiomyocytes from CaMKIIδ^Met281/282^, CaMKIIδ^+/+^ and redox-dead CaMKIIδ^Val281/282^ mice (Fig. 5). Again, this reduction was most likely due to significantly increased spontaneous diastolic SR Ca loss in cardiomyocytes isolated from all aforementioned genotypes (Fig. 6). Vice versa, cardiomyocytes from long-term DOX-treated CaMKIIδ^−/−^ mice revealed preserved SR Ca load, with no detectable Ca leak. Pharmacological inhibition of CaMKII using AIP restored SR Ca content and reduced SR Ca loss in myocytes from CaMKIIδ^Met281/282^, CaMKIIδ^+/+^ and redox-dead CaMKIIδ^Val281/282^ mice following in-vivo DOX treatment. These functional findings were mirrored at the protein level, where long-term DOX treatment led to increased CaMKIIδ activity (by autophosphorylation) in myocytes from CaMKIIδ^+/+^, CaMKIIδ^Met281/282^ and redox-dead CaMKIIδ^Val281/282^ mice. This was associated with CaMKIIδ-dependent RyR2-hyperphosphorylation at pS2814, but not with PKA-dependent RyR2-hyperphophorylation at pS2809 in all of these genetic strains (Fig. 7). Importantly, DOX-induced RyR2-hyperphosphorylation at pS2814 was not observed in cardiomyocytes from CaMKIIδ^−/−^ mice following long-term DOX treatment in-vivo. CaMKII oxidation, however, was unaltered between groups despite a persistently increased prooxidant milieu in both WT lines that was attenuated in myocytes from CaMKIIδ^Val281/282^ and CaMKIIδ^−/−^ mice (**Fig. S7**). Increased DOX-related ROS formation was further associated with increased malondialdehyde (MDA) levels as a biochemical indication of increased oxidative stress, which was in line with decreased SOD activity in all groups except for CaMKIIδ^−/−^ (see **Fig. S8**). Neither Catalase expression, SERCA2a expression nor PLB expression or CaMKII-dependent phosphorylation were altered upon DOX treatment in myocytes from CaMKIIδ^Met281/282^, CaMKIIδ^+/+^, CaMKIIδ^Val281/282^ or CaMKIIδ^−/−^ mice following long-term DOX-treatment (Table 8 **a&b**).

**Table 8:** Protein expression and phosphorylation status of CaMKIIδ^Met281/282^, CaMKIIδ^Val281/282^ cardiomyocytes, CaMKIIδ^+/+^ and CaMKIIδ^−/−^ cardiomyocytes.

Table 8a								
Protein	CaMKIIδ^Met281/282^				CaMKIIδ^Val281/282^			
	Vehicle	n	DOX	n	Vehicle	n	DOX	n
CaMKIIδ	1.000 ± 0.151	7	0.932 ± 0.159	7	0.786 ± 0.113	4	0.584 ± 0.066	6
pT287/CaMKIIδ	1.000 ± 0.232	7	**1.257** **±** **0.067**^a^	7	0.912 ± 0.070	4	**1.344** **±** **0.088**^a^	6
oxCaMKII	1.000 ± 0.344	5	0.911 ± 0.341	5	1.095 ± 0.349	4	1.020 ± 0.314	4
PLB	1.000 ± 0.057	8	1.172 ± 0.100	9	0.882 ± 0.059	5	0.972 ± 0.051	6
pT17/PLB	1.000 ± 0.091	8	0.783 ± 0.066	9	0.948 ± 0.054	5	1.042 ± 0.124	6
RyR2	1.000 ± 0.180	8	1.043 ± 0.100	9	0.567 ± 0.090	5	0.769 ± 0.094	8
pS2814/RyR2	1.000 ± 0.064	8	**1.415** **±** **0.138**^a^	9	1.187 ± 0.057	5	**1.464** **±** **0.076**^a^	8
pS2809/RyR2	1.000 ± 0.083	6	0.940 ± 0.052	6	0.936 ± 0.015	6	1.046 ± 0.090	6
SERCA2a	1.000 ± 0.148	8	1.086 ± 0.134	9	0.880 ± 0.055	5	0.973 ± 0.129	6
Catalase	1.000 ± 0.166	6	1.036 ± 0.188	6	1.057 ± 0.092	6	1.117 ± 0.117	6


Table 8b								
Protein	CaMKIIδ^+/+^				CaMKIIδ^−/−^			
	Vehicle	n	DOX	n	Vehicle	n	DOX	n
CaMKIIδ	1.000 ± 0.045	5	1.078 ± 0.032	6	n.d	6	n.d	6
pT287/CaMKIIδ	1.000 ± 0.137	5	**1.850** **±** **0.265**^a^	6	n.d.	6	n.d.	6
oxCaMKII	1.000 ± 0.2655	5	1.464 ± 0.276	6	n.d	6	n.d	6
PLB	1.000 ± 0.097	9	0.998 ± 0.095	8	0.853 ± 0.244	7	0.764 ± 0.230	7
pT17/PLB	1.000 ± 0.012	9	1.065 ± 0.034	8	0.981 ± 0.186	7	1.071 ± 0.190	7
RyR2	1.000 ± 0.104	9	0.944 ± 0.124	8	1.178 ± 0.169	8	1.105 ± 0.135	7
pS2814/RyR2	1.000 ± 0.052	9	**1.177** **±** **0.037**^a^	8	0.993 ± 0.059	8	**1.006** **±** **0.058**^b^	7
pS2809/RyR2	1.000 ± 0.135	6	0.932 ± 0.106	6	0.935 ± 0.103	5	0.948 ± 0.085	5
SERCA2a	1.000 ± 0.076	17	0.989 ± 0.094	18	1.040 ± 0.175	9	1.041 ± 0.172	9
Catalase	1.000 ± 0.064	6	0.944 ± 0.056	6	0.952 ± 0.079	6	0.830 ± 0.095	6

CaMKIIδ^Met281/282^: wildtype; CaMKIIδ^Val281/282^: redox-dead; CaMKIIδ^+/+^: wildtype; CaMKIIδ^−/−^: genetic CaMKIIδ deletion; CaMKIIδ: Calcium/Calmodulin-dependent protein kinase II δ isoform; pT287/CaMKIIδ: CaMKII autophosphorylation site; oxCaMKII: oxidative-activated CaMKII; PLB: phospholamban; pT17/PLB: CaMKII-specific PLB phosphorylation site; RyR2: ryanodine receptor; pS2814/RyR2; CaMKII-specific RyR2 phosphorylation site; pS2809/RyR2: CaMKII- and PKA-RyR2 phosphorylation site; SERCA2a: SR Ca ATPase.

a Indicates significance vs. vehicle;

b Indicates significance between genotypes using Mann-Whitney-U test or unpaired Student's t-test. n = mice.

**Fig. 8:**
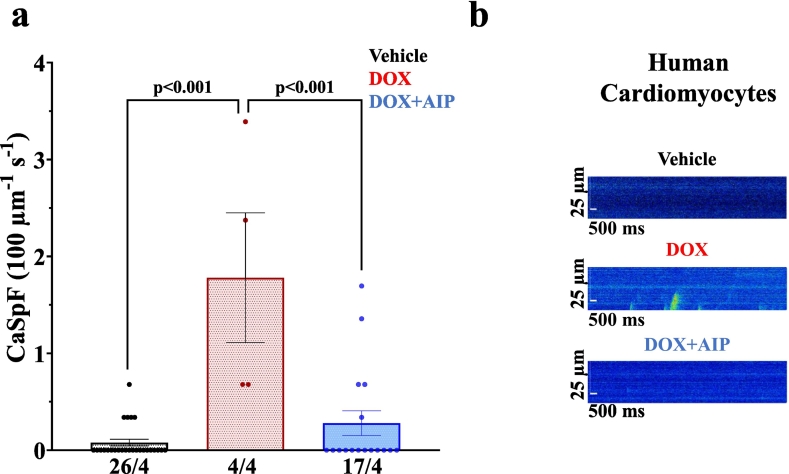
CaMKII mediates SR Ca loss in DOX exposed ventricular human cardiomyocytes. **a-**DOX exposed ventricular human cardiomyocytes show increased diastolic calcium spark frequency (CaSpF) as compared to vehicle-exposed cardiomyocytes. Pharmacological CaMKII inhibition using AIP prevents DOX-induced CaSpF. **b**-Original line-scans of Fluo-4 AM loaded human ventricular cardiomyocytes. *P*-values were calculated using One-Way-ANOVA combined with Holm-Sidak post-hoc test. n = cells/human samples. CaSpF: calcium spark frequency; DOX: doxorubicin.

In a final step, we investigated whether pharmacological inhibition of CaMKII could effectively reduce DOX-related SR Ca leakage in human tissue as well. As depicted in Fig. 8, acute DOX exposure induced significant cardiotoxicity in ventricular human cardiomyocytes, as evidenced by an increase in SR Ca leakage. This effect was markedly inhibited by the CaMKII inhibitor AIP, suggesting that DOX-induced and CaMKIIδ-mediated SR Ca loss is most likely present in human pathology as well, thus presenting a potential therapeutic target.

## 4 Discussion

Our study demonstrates that persistently increased CaMKIIδ autophosphorylation is a key factor during the development of DICM that causally contributes to pathologic SR Ca loss in-vitro and to contractile dysfunction in vivo.

### 4.1 CaMKIIδ autophosphorylation mediates pathologic SR Ca loss and contractile dysfunction in DICM

The most evident feature of DICM with respect to intracellular Ca handling is the depletion of SR Ca load, which is paralleled by LV dysfunction in the long-term course of DICM. SR Ca load is regulated by RyR2-dependent SR Ca *release/loss*, as well as by SERCA2a/PLB-regulated SR Ca *reuptake*. In our model, DOX-related SR Ca depletion most likely results from increased diastolic SR Ca *loss*, mediated by “leaky” RyR2 [14]. “Leaky” RyR2s are a consequence of various post-translational modifications, including hyperphosphorylation, oxidation and glycosylation [14,20,24]. CaMKIIδ is well known to mediate hyperphosphorylation of the RyR2 at its specific phosphorylation site pS2814, which leads to increased RyR2 opening and diastolic SR Ca loss [12,23]. Here, we observed CaMKIIδ-dependent hyperphosphorylation of the RyR2 at pS2814 both in the *acute* and in the *chronic* stage of DICM. It is important to emphasize that DOX-related hyperphosphorylation of the RyR2 at pS2814 was always then observed when CaMKIIδ autophosphorylation was increased. However, redox-dead CaMKIIδ^Val281/282^ myocytes were not protected from DOX-related hyperphosphorylation of the RyR2, because this genetic line still demonstrated robust CaMKIIδ autophosphorylation at pT287. From a mechanistic point of view, this finding suggests a signaling event independent of CaMKIIδ oxidation that alone is enough to activate CaMKIIδ by autophosphorylation.

Our observation that Dantrolene prevents CaMKIIδ autophosphorylation suggests that initial destabilization of the RyR2 and subsequent Ca release from the SR may precede CaMKIIδ autophosphorylation (via Ca/CaM), which would be in line with a previous report from Nakamura et al. [22], in which the authors suggested that Dantrolene serves as a potential agent to treat DICM. While we did not assess the distinct mechanisms of DOX-dependent RyR2 modification here, our data supports the idea that DOX-related mitochondrial ROS formation may be causally involved, because Mito-TEMPO largely prevented SR Ca loss and CaMKIIδ autophosphorylation in the acute setting as well [26].

Most importantly, pathologic RyR2 hyperphosphorylation and subsequently hampered intracellular Ca handling were absent following pharmacological inhibition of CaMKII (using AIP) or by genetic deletion of CaMKIIδ (in CaMKIIδ^−/−^ cardiomyocytes). Our observation that S2814A cardiomyocytes were also largely protected from DOX-mediated diastolic SR Ca loss despite hyperactivation of CaMKIIδ further corroborates our conclusion that CaMKIIδ-dependent hyperphosphorylation of serine-2814 at the RyR2 is the distinct cause of DOX-dependent pathologic SR Ca loss [1,8].

Besides increased SR Ca loss, impaired SR Ca reuptake may have also contributed to SR Ca depletion. Indeed, in our model, diastolic decay kinetics of the systolic Ca transient were prolonged (suggesting impaired SR Ca reuptake), while SERCA2a expression was unaltered, which generally agrees with Llach et al. [18]. Interestingly, SERCA2a is target of oxidative-dependent modifications as well [14]. Thus, DOX-derived ROS might result in oxidation of SERCA2a, contributing to prolonged relaxation kinetics and impaired SR Ca loading (as observed in both acute and chronic DICM). However, we did not detect any DOX-related alterations in CaMKII-dependent phosphorylation of the SERCA2a regulator protein PLB, or with respect to PLB/SERCA2a ratio. Instead, CaMKII inhibition (that usually *prolongs* SR Ca reuptake) led to significantly *accelerated* diastolic Ca elimination here (i.e. faster relaxation kinetics of the Ca transient), so that we interpret the observed prolongation of diastolic Ca elimination upon DOX as a sign of increased diastolic SR Ca *loss* rather than of slowed diastolic SR Ca *uptake*. However, the almost complete normalization of relaxation kinetics upon CaMKII inhibition suggests a predominant role for the CaMKIIδ-dependent SR Ca leak with respect to prolonged Ca transient decay, which further underscores its relevance for impaired Ca handling in the context of DOX.

The relevance of CaMKIIδ-dependent RyR2 hyperphosphorylation and CaMKIIδ-dependent SR Ca loss in DICM is further highlighted by the fact that *only* full genetic deletion of CaMKIIδ (i.e. in CaMKIIδ^−/−^ mice) was capable of preserving LV function upon long-term DOX treatment in-vivo. Redox-dead CaMKIIδ^Val281/282^ mice, however, still demonstrated robust CaMKIIδ-dependent RyR2 hyperphosphorylation and CaMKIIδ-dependent SR Ca leakage and were accordingly *not* protected from LV dysfunction in DICM. Thus, we believe that the observed protection of LV function in CaMKIIδ^−/−^ mice as compared to CaMKIIδ^Val281/282^ animals might results from preserved Ca handling in CaMKIIδ^−/−^ mice as a consequence of absent SR Ca loss and maintained SR Ca load.

Since we observed that DOX induces SR Ca loss in human ventricular myocytes in a CaMKII-dependent manner as well, we suggest that therapeutic strategies targeting CaMKIIδ autophosphorylation may hold promise for improving the care of DICM in human pathology as well.

### 4.2 Pathophysiological implications and clinical perspectives

Our data suggest that persistently increased CaMKIIδ autophosphorylation plays a significant role in DICM through the induction of pathologic SR Ca loss (see **graphical abstract**). Stabilization of the RyR2 using Dantrolene as well as ROS scavenging by Mito-TEMPO equally prevented DOX-related CaMKIIδ activation so that we interpret our data in a way that initial DOX-related RyR2 modification (most likely by oxidation, which we did not assess here) appears to cause an initial “Ca pulse” from the SR that is needed to promote CaMKIIδ hyperactivation by Ca/CaM, which in turn further amplifies Ca loss from the SR. Such a “trigger-sustainer” mechanism might be also relevant in other cardiac pathologies involving increased oxidative stress and CaMKIIδ activation (e.g. ischemia-reperfusion). Therefore, pursuing strategies to reduce CaMKIIδ autophosphorylation could be beneficial in the treatment of DICM. Interestingly, CaMKII is also centrally involved in cancer progression such as in breast cancer [29]. Thus, therapeutic inhibition of CaMKII in breast cancer patients suffering from DICM might serve as dual therapeutic approach, maintaining cardiac function upon DOX treatment while simultaneously attenuating cancer progression. Future strategies targeting the elimination of CaMKIIδ autophosphorylation in both cardiac and malignant tissue - such as by small-molecule inhibitors or by using CRISPR-Cas9 base editing – may prove effective in attenuating DICM in breast cancer patients, but this admittedly speculative hypothesis would have to be tested in preclinical models first [16].

### 4.3 Limitations

The present data mainly come from animal experiments, so any transfer to human pathology should be made with caution. Nevertheless, our findings in human ventricular cardiomyocytes suggest that CaMKII may play a relevant cross-species role in mediating DICM. We cannot exclude that CaMKII in non-cardiomyocyte cell lines (e.g. fibroblasts and immune cells) might be involved in the progression of DICM and might have been affected by our systemic attempt to inhibit CaMKII by pharmacological and genetic measures. Thus, further studies would be necessary to investigate the impact of CaMKII in non-cardiomyocyte cells for the progression of DICM. In addition, further studies are necessary to evaluate the potential impact of other CaMKII isoforms (e.g. CaMKIIγ) in DICM, and to expand the observational period beyond the time frame of 12 weeks as investigated here.

## CRediT authorship contribution statement

**Anna-Lena Feder:** Data curation, Formal analysis, Investigation, Methodology, Software, Validation, Visualization, Writing – original draft, Writing – review & editing. **Daniel Tarnowski:** Data curation, Formal analysis, Investigation, Methodology, Project administration, Resources, Software, Validation, Visualization, Writing – original draft, Writing – review & editing. **Anna-Maria Pfützenreuter:** Data curation, Investigation, Methodology. **Maria Johanna Baier:** Methodology. **Julian Mustroph:** Resources. **Maithily S. Nanadikar:** Methodology. **Dörthe M. Katschinski:** Methodology. **Lars Siegfried Maier:** Funding acquisition, Project administration, Resources, Supervision. **Can Martin Sag:** Conceptualization, Data curation, Formal analysis, Funding acquisition, Investigation, Methodology, Project administration, Resources, Software, Supervision, Validation, Visualization, Writing – original draft, Writing – review & editing.

## Declaration of competing interest

The authors have declared that no conflict of interest exists. Generative artificial intelligence was not used in this work.

## Appendix A Supplementary data

**Supplemental Fig. 1**: CaMKIIδ autophosphorylation prolongs Ca transient decay. **a**-CaMKIIδ^Met281/282^ and CaMKIIδ^Val281/282^ as well as CaMKIIδ^+/+^ cardiomyocytes (**b**) show impaired Ca transient decay kinetics upon acute DOX exposure, which is absent upon pharmacological and genetic CaMKII inhibition (in CaMKIIδ^−/−^). **c**-Prolonged Ca transient decay in CaMKIIδ^Met281/282^, CaMKIIδ^Val281/282^, and CaMKIIδ^+/+^ cardiomyocytes (**d**) in DICM, but not upon pharmacological or genetic inhibition of CaMKII. *P*-values were calculated using Kruskal-Wallis test. n = cells/mice. DOX: doxorubicin. **Supplemental Fig. 2**: Increased cytoplasmic ROS levels depend on mitochondrial ROS formation upon acute DOX exposure. Supplemental Fig. 2 shows increased cytoplasmic ROS formation in DOX-exposed WT- (**a**), CaMKIIδ^Val281/282^ (**b**) and CaMKIIδ^−/−^ cardiomyocytes (**c**), which is reduced by preincubation with the ROS scavenger Mito-TEMPO. *P*-values were calculated using TW-RM ANOVA combined with Holm-Sidak post-hoc test. n = mice. DOX: doxorubicin. **Supplemental Fig. 3**: ROS scavenging and inhibition of the RyR2 attenuates DOX-mediated SR Ca loss. **a**-Original western blots and mean data (**b**) show increased CaMKIIδ autophosphorylation in WT cardiomyocytes upon acute DOX exposure, which is prevented by Mito-TEMPO and Dantrolene. **c&d**-Mito-TEMPO and Dantrolene prevent CaMKIIδ-mediated hyperphosphorylation of the RyR2 at pS2814 upon acute DOX exposure. **e**-Mean data illustrate increased CaSpF in WT-, and CaMKIIδ^Val281/282^ cardiomyocytes upon acute DOX exposure, which is prevented by Mito-TEMPO and Dantrolene and fully absent in CaMKIIδ^−/−^ cardiomyocytes. **f**-Original confocal line scan images of Fluo-4 AM loaded WT-, CaMKIIδ^Val281/282^ and CaMKIIδ^−/−^ cardiomyocytes. *P*-values were calculated using Kruskal-Wallis test (b, e) or One-Way-ANOVA combined with Holm-Sidak post-hoc test (d). n = mice. CaSpF: Ca spark frequency; DOX: doxorubicin; pS2814: CaMKII-specific RyR2 phosphorylation site at serine-2814; pT287: CaMKII autophosphorylation site at threonine-287; RyR2: ryanodine receptor. **Supplemental Fig. 4**: S2814A cardiomyocytes are protected from DOX-induced SR Ca loss despite CaMKIIδ oxidation and hyperphosphorylation. Original western blots (**a**) depict hyperactivation of CaMKIIδ by oxidation (**b**) and autophosphorylation at threonine-287 (**c**) in WT and S2814A cardiomyocytes. **d**-Mean data show increased Ca spark frequency in WT cardiomyocytes upon DOX, which is absent in by S2814A cardiomyocytes. **e**-Original confocal line scan images from Fluo-4 AM loaded WT-, and S2814A cardiomyocytes acutely exposed to DOX. *P*-Values were calculated using unpaired Student's *t*-test (b,c) or Mann-Whitney-*U* test (d). n = mice. CaSpF: calcium spark frequency; DOX: doxorubicin; pT287: CaMKII autophosphorylation site at threonine-287. **Supplemental Fig. 5**: DOX treatment reduces body weight in DICM. Supplemental **Fig. 4a** and **b** depict decreased body weight of DOX-treated CaMKIIδ^Met281/282^, CaMKIIδ^Val281/282^, CaMKIIδ^+/+^ and CaMKIIδ^−/−^ mice as compared to vehicle-treated mice after 12 weeks. All genetic strains exhibited a similar heart weight/body weight ratio, indicating consistent body weight changes across all strains following treatment (**c, d**). *P*-Values were calculated using unpaired Student's t-test (a,b,d) or Mann-Whitney-U test (c). n = mice. DOX: doxorubicin. **Supplemental Fig. 6**: Impaired survival upon DOX treatment. **a**-DOX-treated CaMKIIδ^Met281/282^ mice demonstrated a significantly reduced overall survival compared to vehicle-treated controls. **b**-In DOX-treated CaMKIIδ^Val281/282^ mice, overall survival showed a numerical decrease compared to vehicle-treated controls. **c&d**-Both CaMKIIδ^+/+^ and CaMKIIδ^−/−^ mice demonstrated a trend toward decreased overall survival following DOX treatment. *P*-values were calculated using log-rank test. n = mice. DOX: doxorubicin. **Supplemental Fig. 7**: Cytoplasmic ROS formation in DICM is attenuated in CaMKIIδ^Val281/282^ and CaMKIIδ^−/−^ myocytes. **a-**Long-term DOX treatment results in increased formation of cytoplasmic reactive oxygen species (ROS) in CaMKIIδ^Met281/282^ cardiomyocytes, which is attenuated in CaMKIIδ^Val281/282^ cardiomyocytes. **b**-Original scans of CellROX Orange-loaded CaMKIIδ^Met281/282^ and CaMKIIδ^Val281/282^ cardiomyocytes upon long-term DOX treatment. **c**-Genetic deletion of CaMKII**δ** attenuates DOX-related cytoplasmic ROS formation. **d**-Original scans of CellROX Orange-loaded CaMKIIδ^+/+^ and CaMKIIδ^−/−^ cardiomyocytes upon long-term DOX treatment. ^⁎^indicates significance using Two-Way-ANOVA for repeated measures (RM) combined with Holm-Sidak post-hoc test. DOX: doxorubicin; ROS: reactive oxygen species. **Supplemental Fig. 8**: SOD activity and MDA levels upon long-term DOX treatment. Increased MDA levels as indication of increased oxidative stress are detected across all genetic strains following DOX treatment (**a,b**). Long-term DOX treatment results in increased WST absorbance as a sign of reduced SOD activity in CaMKIIδ^Met281/282^, CaMKIIδ^Val281/282^ (**c**) and CaMKIIδ^+/+^ cardiomyocytes but is not present in CaMKIIδ^−/−^ cardiomyocytes (**d**). *P*-Values were calculated using unpaired Student's *t*-test (c) Mann-Whitney-*U* test (a,b,d). n = mice. DOX: doxorubicin; MDA: malondialdehyde; WST: tetrazolium. Image 1
