# Supplementary material for: Persistently increased CaMKIIδ autophosphorylation mediates pathologic SR Ca loss in a murine model of Doxorubicin-induced cardiomyopathy
Source: J Mol Cell Cardiol Plus. 2025 Nov 21;15:100828. doi: 10.1016/j.jmccpl.2025.100828 (PMC12721290; doi:10.1016/j.jmccpl.2025.100828)
Supplement: Supplementary file 1 — Supplemental Fig. 1: CaMKIIδ autophosphorylation prolongs Ca transient decay. a-CaMKIIδMet281/282 and CaMKIIδVal281/282 as well as CaMKIIδ+/+ cardiomyocytes (b) show impaired Ca transient decay kinetics upon acute DOX exposure, which is absent upon pharmacological and genetic CaMKII inhibition (in CaMKIIδ−/−). c-Prolonged Ca transient decay in CaMKIIδMet281/282, CaMKIIδVal281/282, and CaMKIIδ+/+ cardiomyocytes (d) in DICM, but not upon pharmacological or genetic inhibition of CaMKII. P-values were calculated using Kruskal-Wallis test. n = cells/mice. DOX: doxorubicin. Supplemental Fig. 2: Increased cytoplasmic ROS levels depend on mitochondrial ROS formation upon acute DOX exposure. Supplemental Fig. 2 shows increased cytoplasmic ROS formation in DOX-exposed WT- (a), CaMKIIδVal281/282 (b) and CaMKIIδ−/− cardiomyocytes (c), which is reduced by preincubation with the ROS scavenger Mito-TEMPO. P-values were calculated using TW-RM ANOVA combined with Holm-Sidak post-hoc test. n = mice. DOX: doxorubicin. Supplemental Fig. 3: ROS scavenging and inhibition of the RyR2 attenuates DOX-mediated SR Ca loss. a-Original western blots and mean data (b) show increased CaMKIIδ autophosphorylation in WT cardiomyocytes upon acute DOX exposure, which is prevented by Mito-TEMPO and Dantrolene. c&d-Mito-TEMPO and Dantrolene prevent CaMKIIδ-mediated hyperphosphorylation of the RyR2 at pS2814 upon acute DOX exposure. e-Mean data illustrate increased CaSpF in WT-, and CaMKIIδVal281/282 cardiomyocytes upon acute DOX exposure, which is prevented by Mito-TEMPO and Dantrolene and fully absent in CaMKIIδ−/− cardiomyocytes. f-Original confocal line scan images of Fluo-4 AM loaded WT-, CaMKIIδVal281/282 and CaMKIIδ−/− cardiomyocytes. P-values were calculated using Kruskal-Wallis test (b, e) or One-Way-ANOVA combined with Holm-Sidak post-hoc test (d). n = mice. CaSpF: Ca spark frequency; DOX: doxorubicin; pS2814: CaMKII-specific RyR2 phosphorylation site at serine-2814; pT287: CaMKII autophosphory [file mmc1.pptx]

## Slide 1
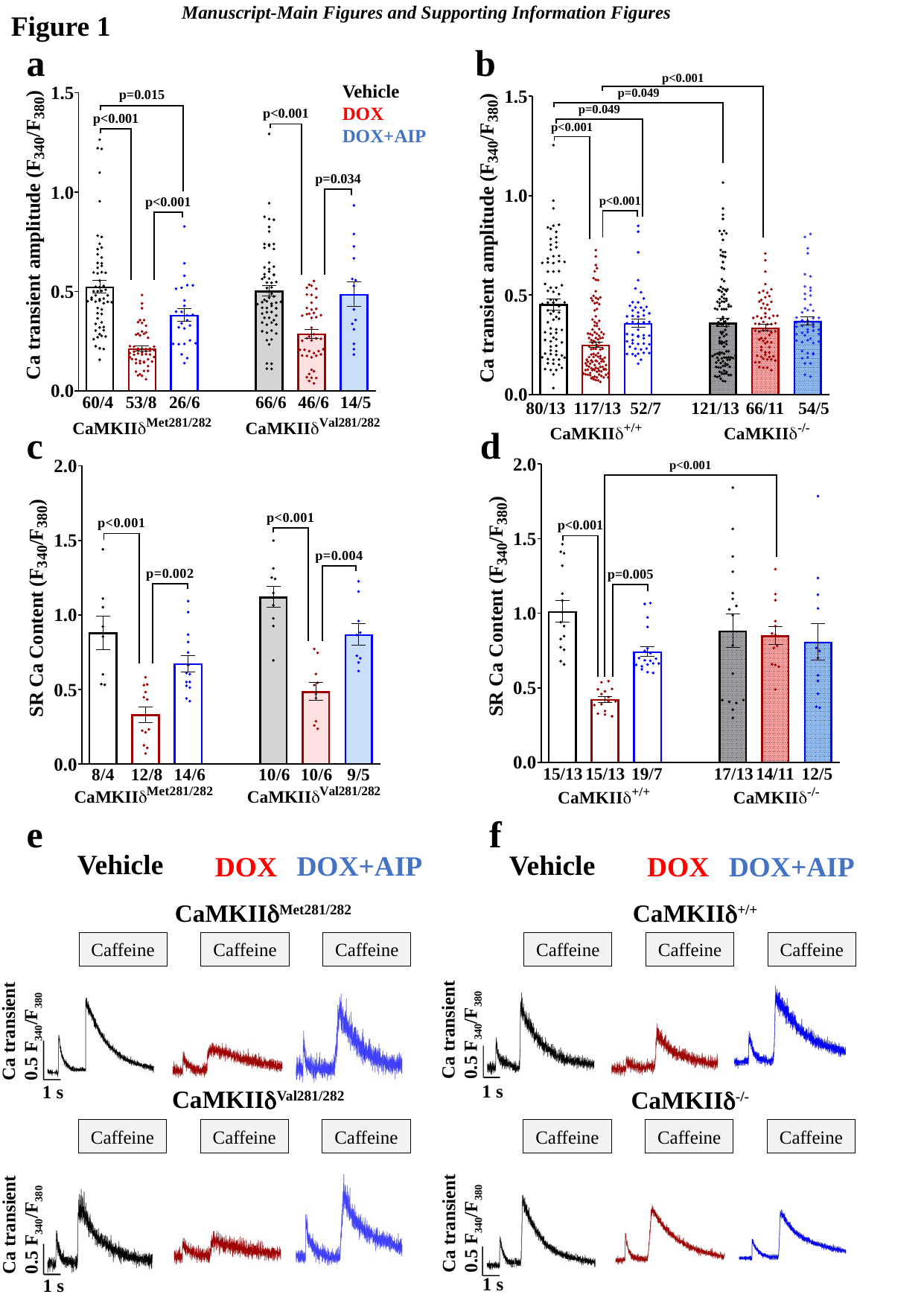

Manuscript-Main Figures and Supporting Information Figures
Figure 1
a
b
Vehicle
DOX
DOX+AIP
d
c
e
f
Vehicle
Vehicle
DOX+AIP
DOX+AIP
DOX
DOX
CaMKIIdMet281/282
CaMKIId+/+
Caffeine
Caffeine
Caffeine
Caffeine
Caffeine
Caffeine
Ca transient
0.5 F340/F380
Ca transient
0.5 F340/F380
1 s
1 s
CaMKIIdVal281/282
CaMKIId-/-
Caffeine
Caffeine
Caffeine
Caffeine
Caffeine
Caffeine
Ca transient
0.5 F340/F380
Ca transient
0.5 F340/F380
1 s
1 s

## Slide 2
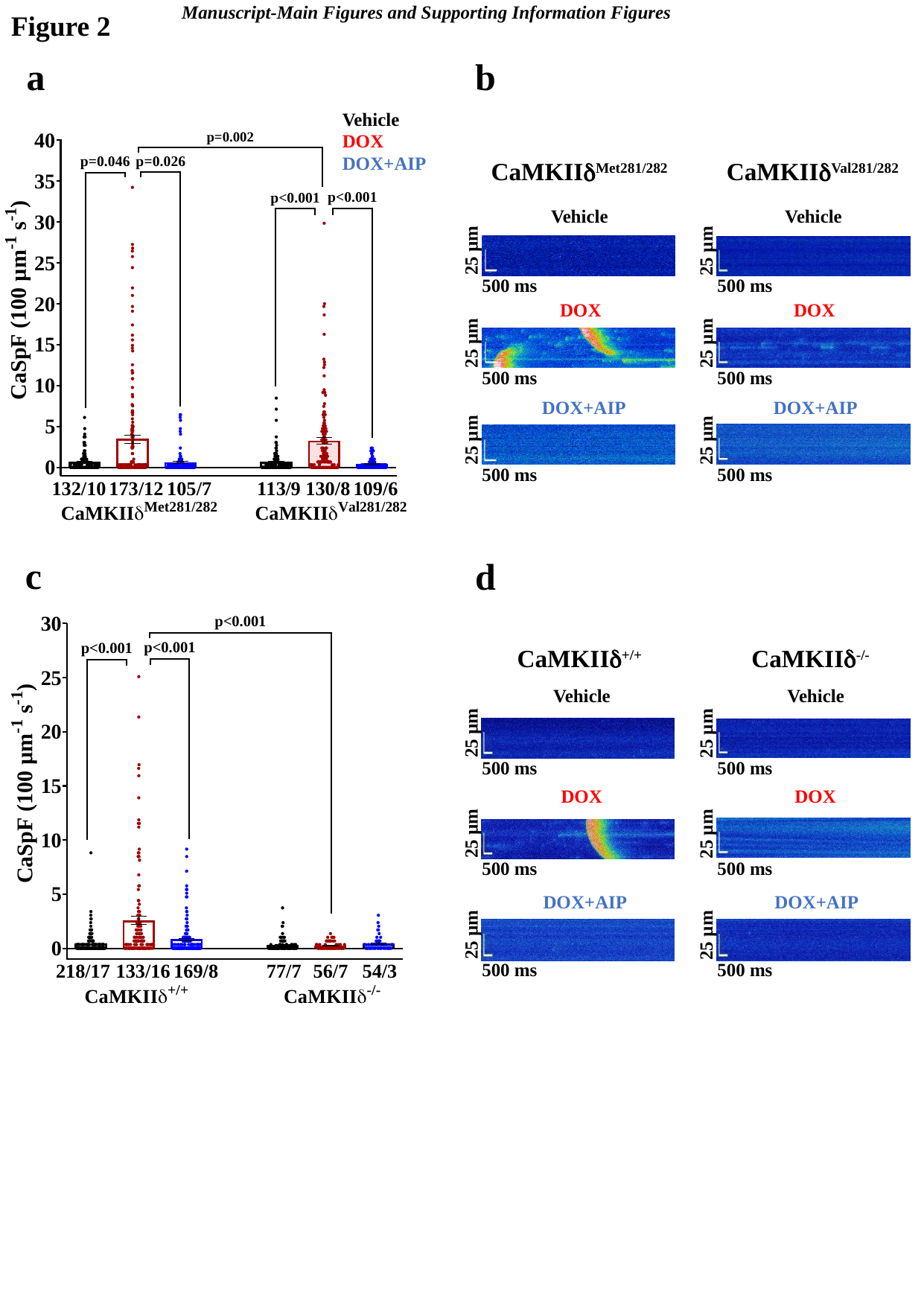

Manuscript-Main Figures and Supporting Information Figures
Figure 2
a
b
Vehicle
DOX
DOX+AIP
CaMKIIdVal281/282
CaMKIIdMet281/282
Vehicle
Vehicle
25 µm
25 µm
500 ms
500 ms
DOX
DOX
25 µm
25 µm
500 ms
500 ms
DOX+AIP
DOX+AIP
25 µm
25 µm
500 ms
500 ms
c
d
CaMKIId-/-
CaMKIId+/+
Vehicle
Vehicle
25 µm
25 µm
500 ms
500 ms
DOX
DOX
25 µm
25 µm
500 ms
500 ms
DOX+AIP
DOX+AIP
25 µm
25 µm
500 ms
500 ms

## Slide 3
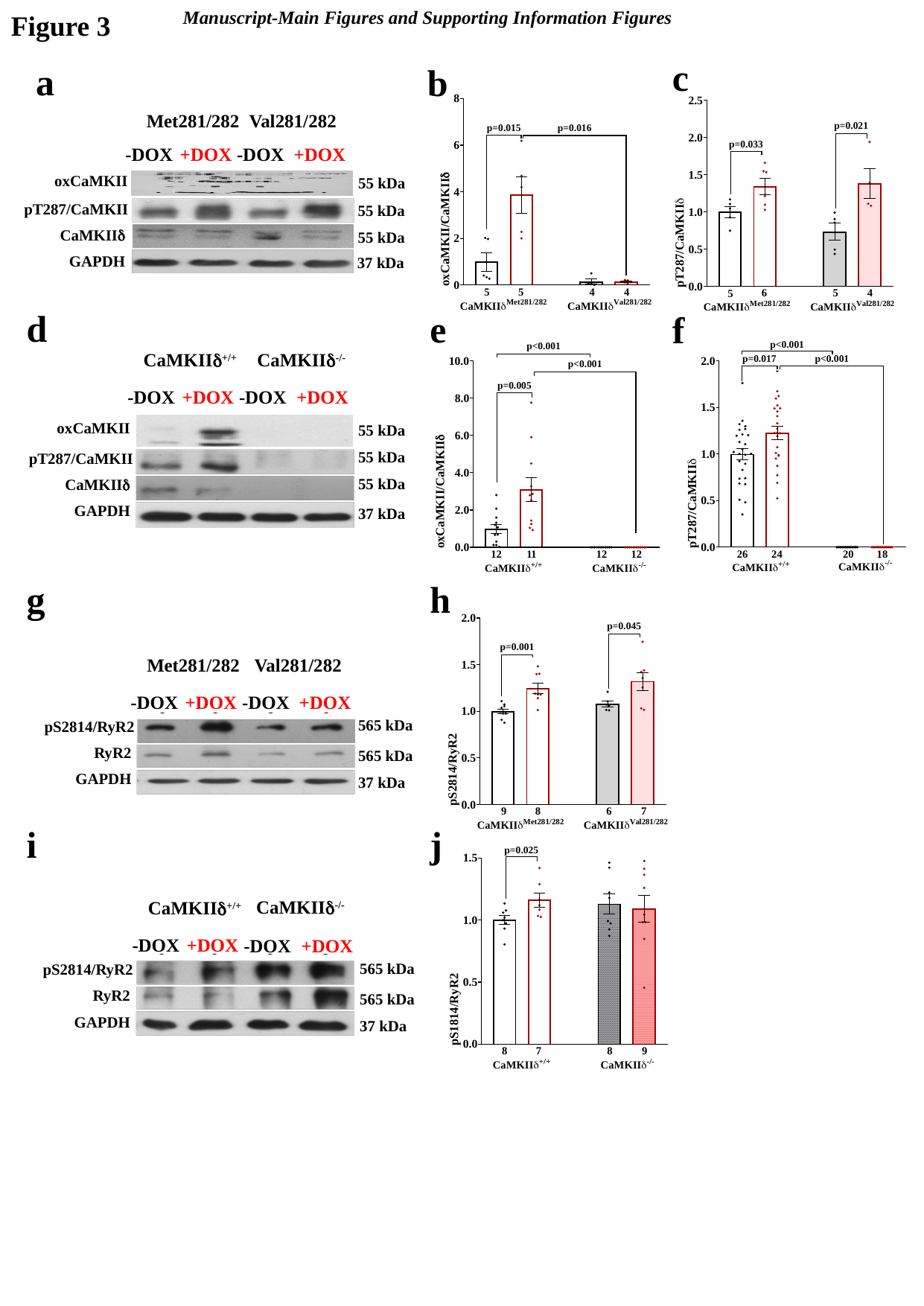

Manuscript-Main Figures and Supporting Information Figures
Figure 3
c
a
b
Val281/282
Met281/282
+DOX
-DOX
+DOX
-DOX
oxCaMKII
55 kDa
pT287/CaMKII
55 kDa
CaMKIId
55 kDa
GAPDH
37 kDa
d
e
f
CaMKIId+/+
CaMKIId-/-
-DOX
+DOX
-DOX
+DOX
oxCaMKII
55 kDa
55 kDa
pT287/CaMKII
55 kDa
CaMKIId
GAPDH
37 kDa
g
h
Val281/282
Met281/282
+DOX
-DOX
+DOX
-DOX
565 kDa
pS2814/RyR2
RyR2
565 kDa
GAPDH
37 kDa
i
j
CaMKIId-/-
CaMKIId+/+
-DOX
+DOX
-DOX
+DOX
565 kDa
pS2814/RyR2
RyR2
565 kDa
GAPDH
37 kDa

## Slide 4
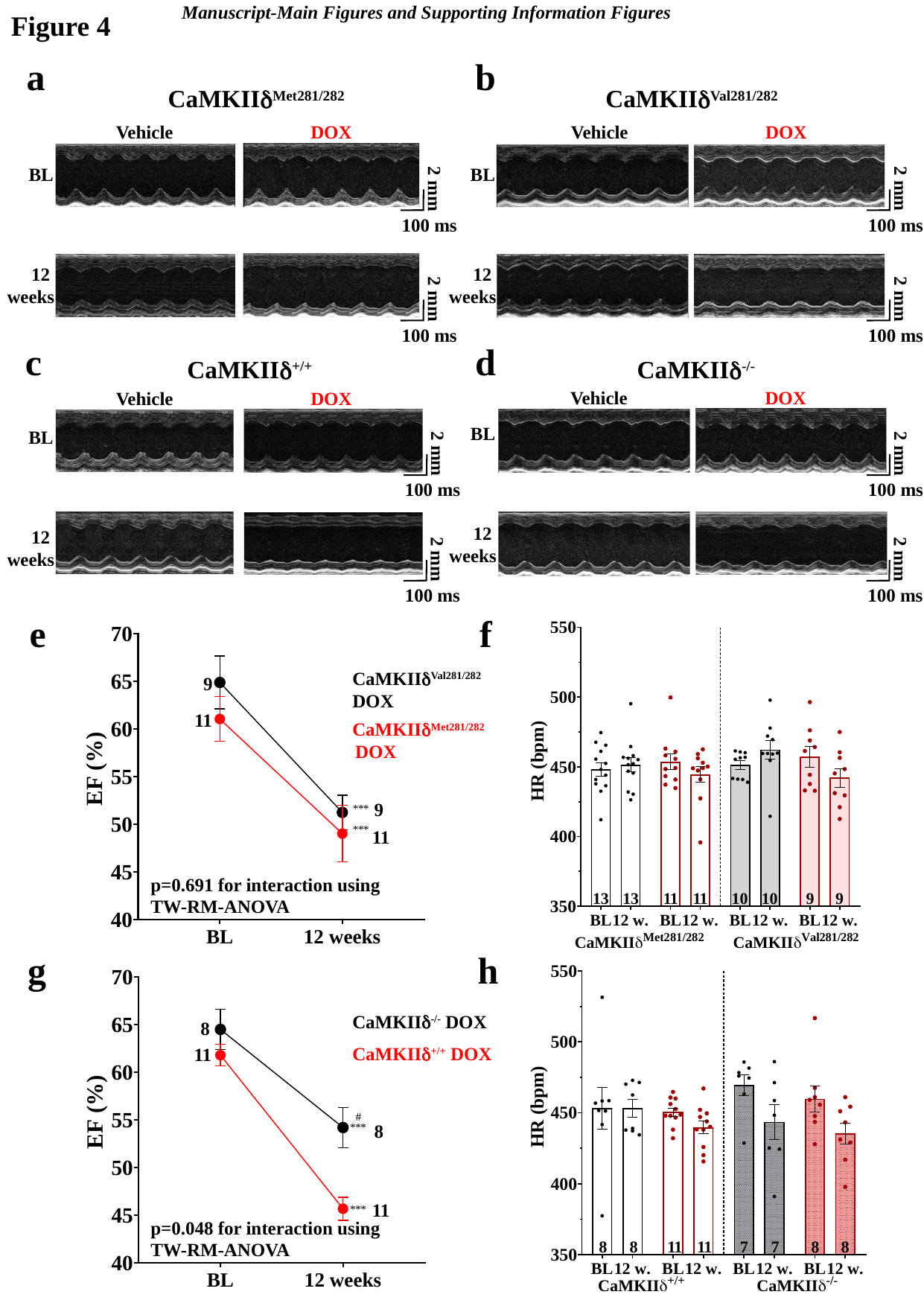

Manuscript-Main Figures and Supporting Information Figures
Figure 4
a
b
CaMKIIdMet281/282
CaMKIIdVal281/282
DOX
Vehicle
DOX
Vehicle
2 mm
100 ms
2 mm
100 ms
BL
BL
12
weeks
12
weeks
2 mm
100 ms
2 mm
100 ms
c
d
CaMKIId+/+
CaMKIId-/-
DOX
Vehicle
DOX
Vehicle
BL
BL
2 mm
100 ms
2 mm
100 ms
12
weeks
12
weeks
2 mm
100 ms
2 mm
100 ms
e
f
CaMKIIdVal281/282 DOX
9
11
CaMKIIdMet281/282
 DOX
9
11
p=0.691 for interaction using
TW-RM-ANOVA
g
h
CaMKIId-/- DOX
8
CaMKIId+/+ DOX
11
8
11
p=0.048 for interaction using
TW-RM-ANOVA

## Slide 5
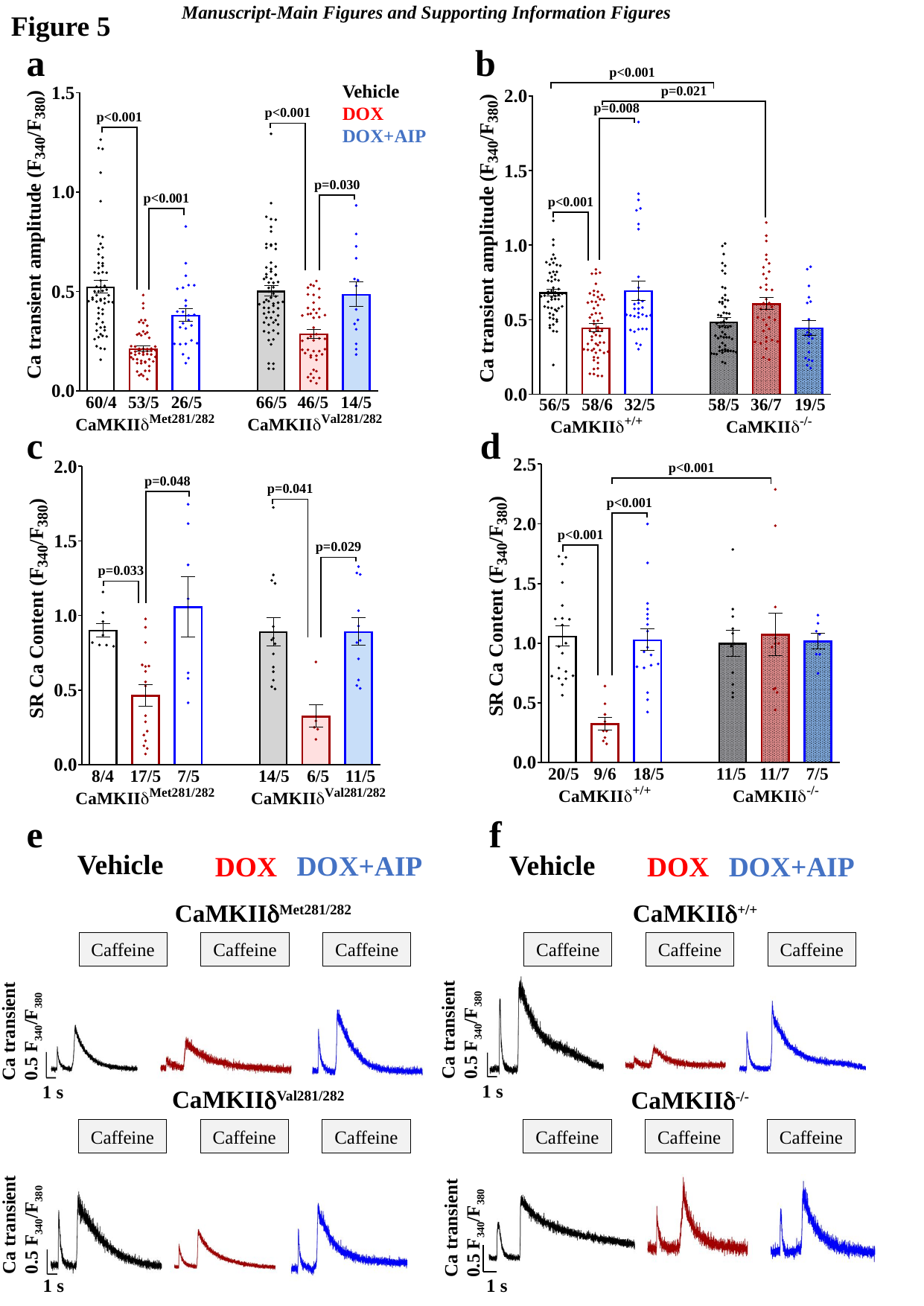

Manuscript-Main Figures and Supporting Information Figures
Figure 5
a
b
Vehicle
DOX
DOX+AIP
d
c
e
f
Vehicle
Vehicle
DOX+AIP
DOX+AIP
DOX
DOX
CaMKIIdMet281/282
CaMKIId+/+
Caffeine
Caffeine
Caffeine
Caffeine
Caffeine
Caffeine
Ca transient
0.5 F340/F380
Ca transient
0.5 F340/F380
1 s
1 s
CaMKIIdVal281/282
CaMKIId-/-
Caffeine
Caffeine
Caffeine
Caffeine
Caffeine
Caffeine
Ca transient
0.5 F340/F380
Ca transient
0.5 F340/F380
1 s
1 s

## Slide 6
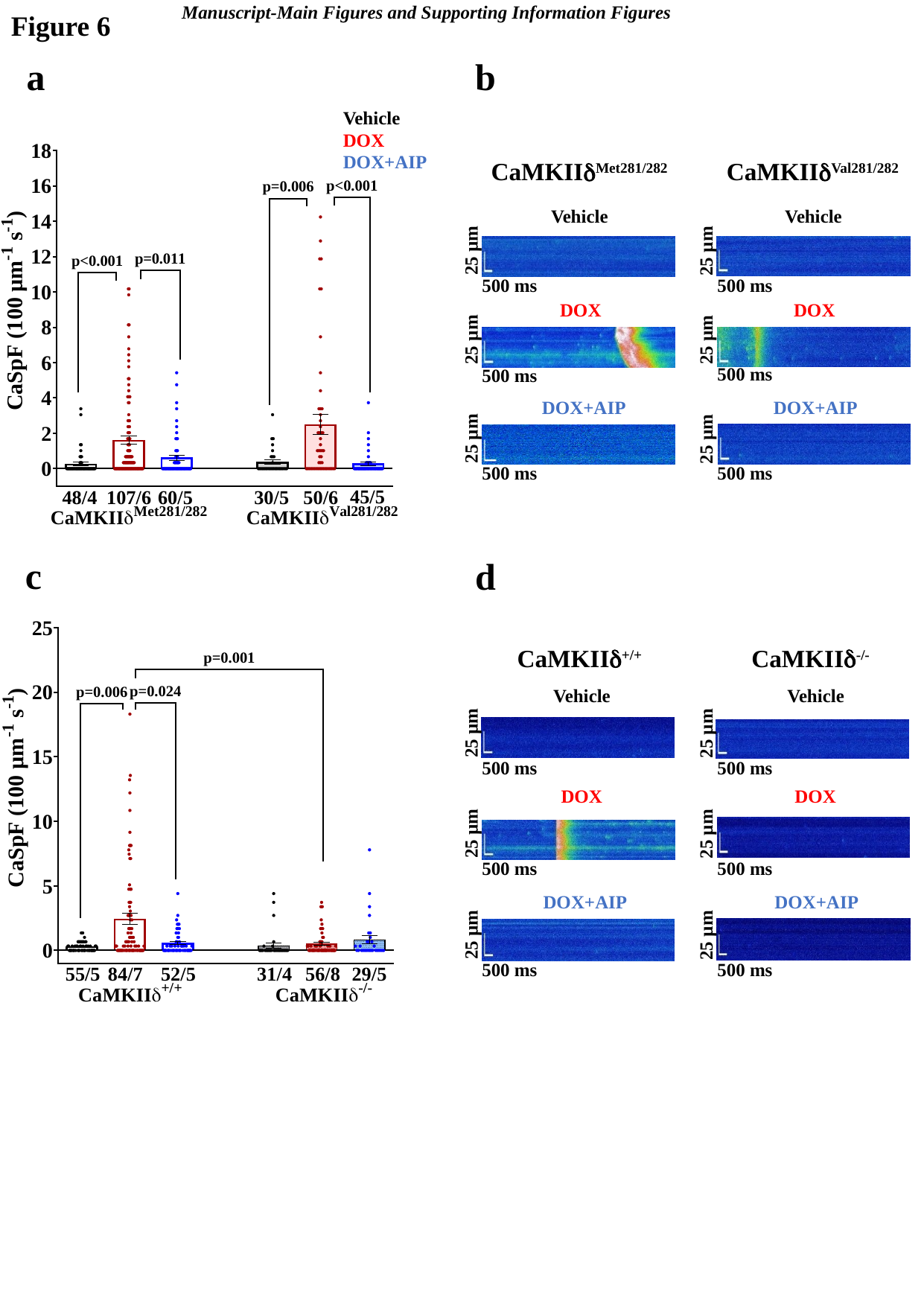

Manuscript-Main Figures and Supporting Information Figures
Figure 6
a
b
Vehicle
DOX
DOX+AIP
CaMKIIdVal281/282
CaMKIIdMet281/282
Vehicle
Vehicle
25 µm
25 µm
500 ms
500 ms
DOX
DOX
25 µm
25 µm
500 ms
500 ms
DOX+AIP
DOX+AIP
25 µm
25 µm
500 ms
500 ms
c
d
CaMKIId-/-
CaMKIId+/+
Vehicle
Vehicle
25 µm
25 µm
500 ms
500 ms
DOX
DOX
25 µm
25 µm
500 ms
500 ms
DOX+AIP
DOX+AIP
25 µm
25 µm
500 ms
500 ms

## Slide 7
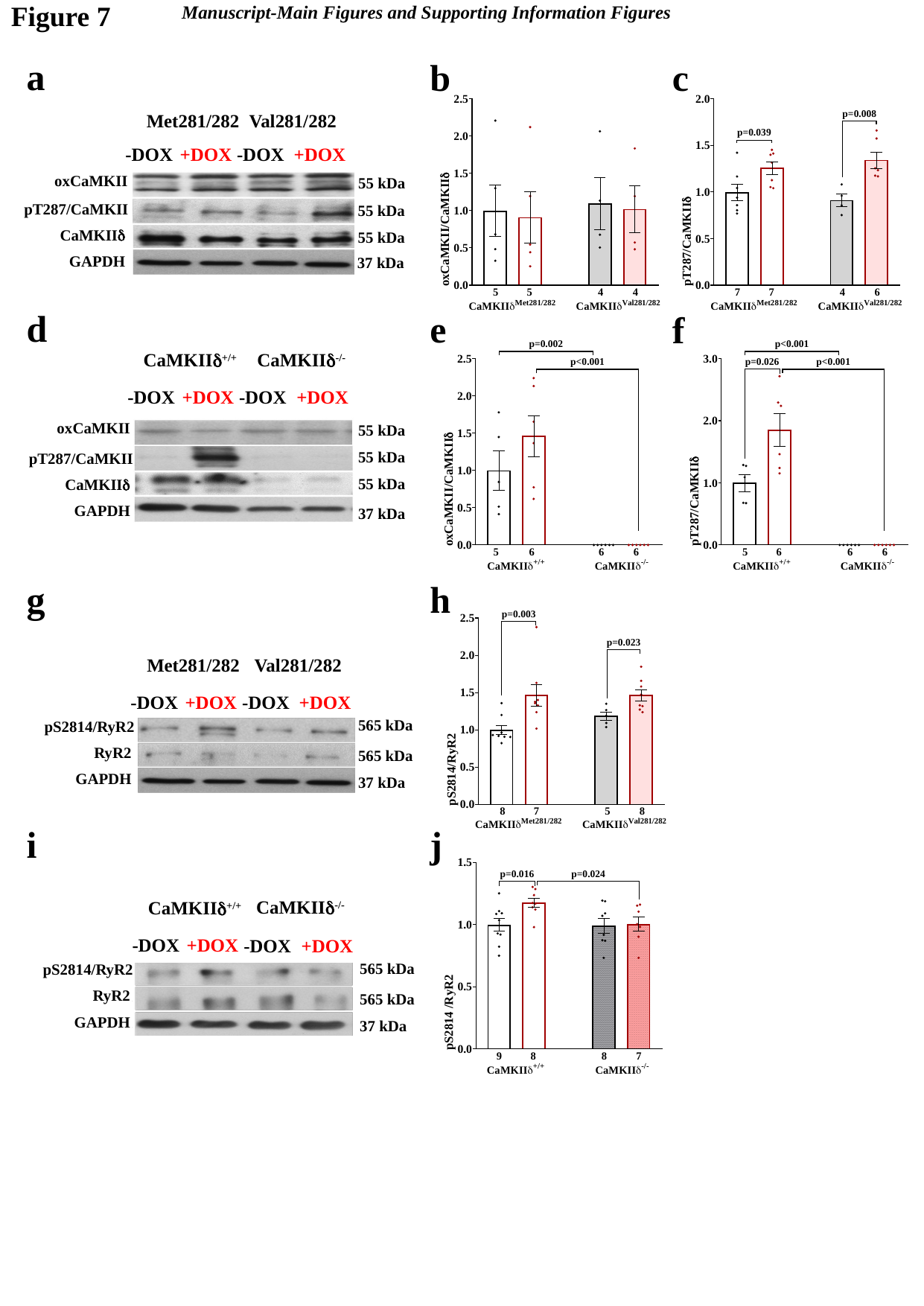

Figure 7
Manuscript-Main Figures and Supporting Information Figures
a
b
c
Val281/282
Met281/282
+DOX
-DOX
+DOX
-DOX
oxCaMKII
55 kDa
pT287/CaMKII
55 kDa
CaMKIId
55 kDa
GAPDH
37 kDa
d
e
f
CaMKIId+/+
CaMKIId-/-
-DOX
+DOX
-DOX
+DOX
oxCaMKII
55 kDa
55 kDa
pT287/CaMKII
55 kDa
CaMKIId
GAPDH
37 kDa
g
h
Val281/282
Met281/282
+DOX
-DOX
+DOX
-DOX
565 kDa
pS2814/RyR2
RyR2
565 kDa
GAPDH
37 kDa
i
j
CaMKIId-/-
CaMKIId+/+
-DOX
+DOX
-DOX
+DOX
565 kDa
pS2814/RyR2
RyR2
565 kDa
GAPDH
37 kDa

## Slide 8
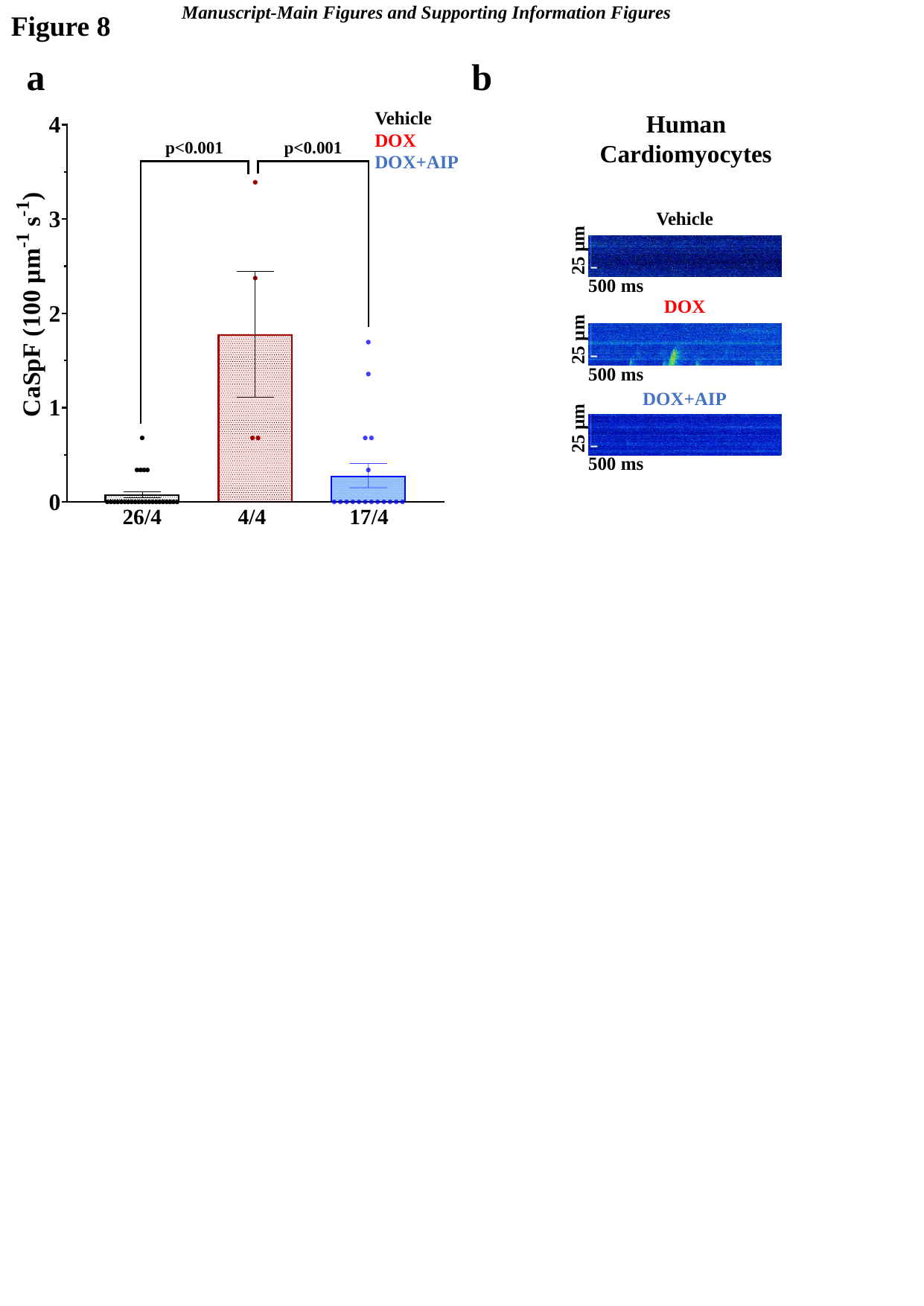

Manuscript-Main Figures and Supporting Information Figures
Figure 8
a
b
Vehicle
DOX
DOX+AIP
Human Cardiomyocytes
Vehicle
25 µm
500 ms
DOX
25 µm
500 ms
DOX+AIP
25 µm
500 ms
